# An (*αβ*)_2_ Architecture of the *Arabidopsis thaliana* tRNA Splicing Endonuclease Complex: Structural Modeling and Evolutionary Analyses

**DOI:** 10.1002/prot.70155

**Published:** 2026-07-09

**Authors:** Mohammad Moniruzzaman, Markus Englert, Kazuhito Akama

**Affiliations:** ^1^ Graduate School of Natural Science & Technology Shimane University Matsue Shimane Japan; ^2^ Independent Researcher Whittlesford UK; ^3^ Department of Life Sciences Shimane University Matsue Shimane Japan

**Keywords:** AlphaFold3, *Arabidopsis thaliana*, ConSurf, MMGBSA, pre‐tRNA, protein–RNA interaction, tRNA splicing endonuclease

## Abstract

The tRNA splicing endonuclease (TSEN) removes introns from precursor tRNAs (pre‐tRNAs) in eukaryotes and archaea, but the architecture and subunit organization of plant TSEN remain poorly defined. Here, we integrated *in planta* bimolecular fluorescence complementation (BiFC) assays with AlphaFold3 (AF3)‐based structural modeling, molecular dynamics simulations, molecular mechanics generalized Born surface area (MMGBSA) decomposition, and ConSurf analysis to characterize the *Arabidopsis thaliana* TSEN complex (AtTSEN), focusing on AtTSEN1, AtTSEN2, and AtTSEN54. Sequence‐ and structure‐based homology searches did not detect a canonical TSEN15 homolog, a structural subunit conserved in yeast and human TSEN. BiFC supported pairwise interactions between AtTSEN54 and the catalytic subunits AtTSEN1 and AtTSEN2, but not between AtTSEN1 and AtTSEN2, suggesting AtTSEN54‐mediated heterodimeric pairing. AF3 multimer modeling predicted a pseudo‐symmetric archaeal‐like (*αβ*)_2_ architecture for AtTSEN rather than a canonical eukaryotic *αβγδ* arrangement. In the AF3 pre‐tRNA‐bound model, AtTSEN54 was positioned near the pre‐tRNA elbow, whereas the intron‐containing anticodon arm was oriented asymmetrically toward the AtTSEN1 catalytic sites. Per‐residue MMGBSA decomposition identified basic protein residues, particularly Arg and Lys, as putative pre‐tRNA‐binding hotspots, many of which corresponded to evolutionarily constrained interface positions across plant, fungal, and metazoan TSEN complexes. Together, these findings support an archaeal‐like (*αβ*)_2_ structural model for AtTSEN and suggest that shared RNA‐binding features are preserved alongside plant‐specific remodeling of TSEN architecture.

## 1 Introduction

Across all domains of life, tRNAs are transcribed as precursors (pre‐tRNAs) that must be processed before entering translation. For intron‐containing pre‐tRNAs, maturation requires precise intron excision and exon ligation. In bacteria, intron removal is mediated by self‐splicing group I introns [1]. In eukaryotes and archaea, by contrast, the initial endonucleolytic cleavage at the splice sites is catalyzed by a dedicated tRNA splicing endonuclease (TSEN) complex [2].

Throughout this manuscript, AtTSEN, HsTSEN, and ScTSEN refer to TSEN complexes from *Arabidopsis thaliana* , human ( *Homo sapiens* ), and yeast ( *Saccharomyces cerevisiae* ), respectively. Unless otherwise specified, “TSEN” refers generically to the tRNA splicing endonuclease complex, whereas TSEN1, TSEN2, TSEN34, TSEN15, and TSEN54 denote homologous subunits across species. Organism‐specific subunits are indicated by a species prefix followed by the subunit number, for example, AtTSEN1.

Archaeal TSENs assemble as homotetramers (*α*_4_), homodimers (*α*′_2_), heterotetramers [(*αβ*)_2_], or fusion‐derived homodimers (*ε*_2_), whereas eukaryotic TSENs characterized to date adopt a heterotetrameric *αβγδ* organization [2, 3, 4]. Archaeal assembly is stabilized by two conserved interface elements: antiparallel *β*–*β* pairing between adjacent *α* subunits and insertion of a negatively charged L10 loop into a basic pocket of the partner subunit. These structural features are retained across all four archaeal assemblies, and the active sites also harbor a conserved Tyr‐His‐Lys catalytic triad [4].

In yeast and human TSEN complexes, the *αβγδ* heterotetramer is composed of the catalytic subunits TSEN2 and TSEN34 together with the structural subunits TSEN15 and TSEN54 [3, 5]. Within HsTSEN, *β*–*β* interfaces pair HsTSEN15 with HsTSEN34 and HsTSEN2 with HsTSEN54, and L10‐loop contacts further stabilize these dimer interfaces [6]. In both systems, TSEN2 and TSEN34 each harbor a conserved Tyr‐His‐Lys catalytic triad and cleave the 5′ and 3′ splice sites of pre‐tRNA, respectively [3, 7]. Yeast two‐hybrid analysis identified ScTSEN2–54 and ScTSEN34–15 as the major interacting pairs in ScTSEN [3]. Consistently, combinatorial co‐expression analyses established TSEN2–54 and TSEN15–34 as functional heterodimers in HsTSEN [8].

The architectural diversity among archaeal TSEN assemblies is linked to variation in pre‐tRNA substrate architecture. The *α*_4_ homotetrameric archaeal enzymes strictly require the canonical bulge–helix–bulge (BHB) motif [9], whereas *α*′_2_, (*αβ*)_2_, and *ε*_2_ variants additionally process bulge–helix–loop (BHL) substrates, in which one splice‐site bulge is replaced by a single‐stranded loop (also termed relaxed BHB) [4, 10, 11]. Eukaryotic TSEN processes both BHB [12, 13] and BHL substrates [7]; in BHL substrates, where the 5′ bulge is absent, cleavage positions are defined relative to the mature tRNA domain rather than the intron motif [14]. At the subunit level, cryo‐electron microscopy (cryo‐EM) structures of the HsTSEN–pre‐tRNA^Arg^ complex showed that TSEN54 forms the principal elbow‐binding interface and positions TSEN2 and TSEN34 at their respective splice sites, whereas TSEN15 contributes to holoenzyme stability and accurate 5′‐site positioning [6, 15, 16].

Despite these advances in yeast and human systems, the architecture and pre‐tRNA recognition mechanism of the *Arabidopsis* TSEN complex (AtTSEN) remain poorly characterized. In *Arabidopsis*, the catalytic subunits AtTSEN1 and AtTSEN2 share ~72% sequence identity and retain the canonical Tyr‐His‐Lys catalytic triads [17]. Intron‐containing pre‐tRNAs in *Arabidopsis* are largely restricted to elongator tRNA^Met^ and tRNA^Tyr^, both of which harbor short introns (11–25 nt) [18, 19, 20, 21]. Splicing assays using wheat germ endonuclease implicate the D‐stem and A‐I helix length as primary determinants of splicing efficiency [22, 23, 24, 25]. However, these studies do not define the quaternary structure, subunit composition, or interaction logic of the AtTSEN complex.

Here, we combined sequence‐ and structure‐based homology searches with experimental and computational analyses to investigate AtTSEN organization. These searches did not detect a canonical plant counterpart of TSEN15. We then used *in planta* bimolecular fluorescence complementation (BiFC) assays to map pairwise subunit interactions, AlphaFold3 (AF3) [26] modeling to predict AtTSEN architecture and pre‐tRNA positioning, and molecular dynamics (MD) simulations with molecular mechanics generalized Born surface area (MMGBSA) binding free‐energy decomposition to identify putative protein hotspot residues at the TSEN–pre‐tRNA interface. ConSurf‐based evolutionary conservation analysis was used to assess whether these hotspots correspond to conserved interface positions. Together, these approaches provide a structural and evolutionary framework for understanding AtTSEN assembly and pre‐tRNA engagement.

## 2 Materials and Methods

### 2.1 Ortholog Identification and Sequence Retrieval

Candidate *Arabidopsis* homologs of yeast TSEN54 were identified by structure‐based homology searches using Foldseek [27] (3Di/AA mode; default parameters; web interface). The AlphaFold monomer model of ScTSEN54 [28] was used as the query. Searches were performed against all available Foldseek database partitions, and the resulting hits were manually inspected for structural plausibility. An equivalent Foldseek search was conducted to identify candidate TSEN15 homologs using the AlphaFold model of ScTSEN15 as the query with the same settings.

To complement the structure‐based approach, sequence‐based homology searches for TSEN15 candidates were performed using BLASTp and PSI‐BLAST (five iterations; inclusion threshold *E*‐value ≤ 0.10; BLOSUM62 substitution matrix) [29] against the NCBI non‐redundant (nr) protein database [30]. Additional searches were performed through the EMBL‐EBI web server with default significance thresholds [31]; ScTSEN15 was used as the query sequence in all searches. Unless otherwise stated, all amino acid sequences were retrieved from the UniProt database [32], and accession numbers are provided in Table S1. Representative TSEN54 multiple sequence alignments are shown in Figures S1 and S2.

### 2.2 BiFC Analysis

BiFC expression constructs were generated using Gateway cloning [33]. The coding sequences of AtTSEN1 and AtTSEN2 were amplified from cDNA templates [17], whereas the AtTSEN54 cDNA clone was obtained from the RIKEN BioResource Center (BRC no. pda20235; Clone ID RAFL19‐91‐H15). Coding sequences were appended with attB recombination sites via two‐step PCR and recombined into the pDONR221 donor vector using BP Clonase II (Thermo Fisher Scientific). Entry clones were verified by Sanger sequencing and then recombined into the appropriate V‐10 Gateway binary destination vectors (pB4nVGW3, pB4GWnV3, pB4cVGW, and pB4GWcV) [34] using LR Clonase II (Thermo Fisher Scientific). Each target protein was cloned into four split‐Venus fusion constructs, enabling eight possible BiFC combinations for each tested protein pair (AtTSEN1–AtTSEN54, AtTSEN2–AtTSEN54, and AtTSEN1–AtTSEN2) (Table S4). Final constructs were confirmed by Sanger sequencing across the Venus–AtTSEN fusion junctions. Primers used for Gateway cloning are listed in Table S2, and plasmids used for BiFC analysis are listed in Table S3.

For microcarrier preparation, 10 mg of tungsten particles were sonicated in 800 μL of Milli‐Q water and pelleted by centrifugation (1000*g*, 1 min). The pellet was washed with 100% ethanol, vacuum‐dried for 5 min, and resuspended in 400 μL of 10× TE buffer. For plasmid coating, 0.5 μg each of the cVenus and nVenus constructs (or empty vector controls) and 1 μg of the pGWB45 plasmid encoding enhanced cyan fluorescent protein (ECFP) [35] were combined with 10 μL of the particle suspension. DNA was precipitated with 3 M sodium acetate (pH 5.2) and 100% ethanol at −20°C for 20 min. Pelleted particles (1000*g*, 1 min) were washed with 70% ethanol, resuspended in 100% ethanol, and stored at 4°C.

Particle delivery was performed using an IDERA GIE‐III system (Tanaka Co. Ltd., Japan). DNA‐coated microcarriers were delivered into the inner epidermal layer of onion ( *Allium cepa* ) bulb segments under 80 kPa vacuum, 0.075 s burst, and 490 kPa (≈71 psi) helium pressure. Bombarded sections were incubated in the dark at 23°C for 14–16 h. Confocal imaging was performed on a Leica TCS SP5 system according to the acquisition settings previously described [36]. All images were acquired as Z‐stacks with acquisition parameters held constant across samples. Mean fluorescence intensities were measured from a single region of interest (ROI) covering the entire cell area for both Venus and ECFP channels. BiFC signal intensity was normalized to the ECFP signal to account for variations in transfection efficiency. Statistical analyses were conducted in JASP version 0.19.1 [37]. Data are presented as mean ± standard error of the mean (SEM; *n* = 4 cells per condition in total). Experiments were performed in three independent trials. Differences between groups were assessed by one‐way analysis of variance (ANOVA) followed by Dunnett's post hoc test, with significance defined as *p ≤ 0.05, **p ≤ 0.01, and ***p ≤ 0.001. Graphs were generated in Microsoft Excel (Office 365, version 16.102) with consistent axis scales across conditions.

### 2.3 Structural Modeling and Electrostatic Surface Potential (ESP) Analysis

Structural models of *Arabidopsis*, human, and yeast TSEN complexes were predicted using AlphaFold3 (AF3) [26], whereas monomer models were retrieved from the AlphaFold Database [28]. Pre‐tRNA sequences were retrieved from GtRNAdb [38] and protein sequences from UniProt [32] (Table S1). The top‐ranked AF3 model (rank 0) was selected for downstream analysis. Model confidence was evaluated using predicted local distance difference test (pLDDT), predicted aligned error (PAE), predicted template modeling score (pTM), and interface predicted template modeling score (ipTM); the corresponding pLDDT‐colored structures, PAE plots, and pTM/ipTM values are summarized in Figure S4.

Structural alignment (five cycles; 2.0 Å cutoff), annotation, and rendering were performed in PyMOL version 3.1.8 [39]. For ESP analysis, models were preprocessed using Dock Prep in UCSF ChimeraX version 1.7.1 [40] and calculated within PyMOL using the Adaptive Poisson–Boltzmann Solver (APBS) GUI plugin [41], with atomic charges and radii assigned via PDB2PQR [42] using the AMBER force field. The linearized Poisson–Boltzmann equation was solved using dielectric constants of 2.0 for protein and 78.0 for solvent (310 K; 0.15 M monovalent ions). Where necessary for visual clarity, extended low‐pLDDT disordered regions were masked in renderings of assembled complex models, whereas short disordered segments were retained; full unmasked models are provided in Figure S4.

### 2.4 MD Simulations

MD simulations of the AtTSEN–pre‐tRNA^Tyr^, HsTSEN–pre‐tRNA^Arg^, and ScTSEN–pre‐tRNA^Phe^ complexes were performed using GROMACS version 2026.1 [43]. Structures were prepared by removing extended N‐terminal disordered low‐pLDDT regions of AtTSEN54 subunits; however, short low‐pLDDT disordered regions in HsTSEN and ScTSEN, as well as all internal loops, were retained to preserve structural continuity. The 5′‐terminal phosphate of each pre‐tRNA chain was removed prior to topology generation to prevent non‐standard terminus errors during AMBER parametrization. System setup, including force‐field assignment with AMBER99SB‐ILDN [44], cubic simulation‐box construction, and TIP3P water‐model assignment [45], was performed using CHAPERONg [46]. Na^+^ and Cl^−^ ions were added using gmx genion to neutralize the system charge and adjust the NaCl concentration to 0.15 M.

Each system was energy‐minimized using the steepest‐descent algorithm for up to 100 000 steps with a maximum force (*F*_max_) of 1000 kJ mol^−1^ nm^−1^. Particle Mesh Ewald (PME) electrostatics were applied with a Fourier spacing of 0.16 nm and a cutoff radius of 1.0 nm [47], alongside a potential‐shift Verlet scheme for van der Waals interactions; these settings were applied throughout equilibration and production simulations. Sequential equilibration was performed under the isothermal–isochoric (NVT) ensemble (500 ps at 310 K; V‐rescale thermostat, *τ* = 0.1 ps) [48], followed by the isothermal–isobaric (NPT) ensemble (1 ns at 310 K and 1 bar [0.1 MPa]; C‐rescale barostat, *τ* = 2.0 ps, compressibility = 4.5 × 10–5 bar^−1^) [49], with position restraints applied to all heavy atoms throughout. Hydrogen bonds were constrained using the Linear Constraint Solver (LINCS) algorithm [50], permitting a 2 fs integration time step. Production MD simulations were then performed for 100 ns under the NPT ensemble without restraints, and coordinates were saved every 10 ps, yielding 10 000 frames.

### 2.5 MMGBSA Decomposition Analysis

Binding free energies (Δ*G*_bind_) and per‐residue energetic contributions (Δ*G*_res_) were calculated using the MMGBSA method as implemented in gmx_MMPBSA version 1.5.0.3 [51]. Calculations were performed over the 70–100 ns trajectory window (3000 frames; interval = 1) after confirming stable convergence by root‐mean‐square deviation (RMSD) analysis. Implicit solvation was modeled using the OBC2 Generalized Born model (igb = 5) [52, 53] with mbondi3 radii (PBRadii = 4), an internal dielectric constant of 10, and a salt concentration of 0.15 M. Total MMGBSA energy components are reported in Table S5. Per‐residue MMGBSA decomposition was performed for all protein and RNA residues within 6 Å of the binding partner. Protein residues with individual Δ*G*_res_ ≤ −1 kcal mol^−1^ were ranked by energetic contribution for each complex. Representative top‐ranked residues are summarized in Table 1, and the complete ranked residue lists are provided in Table S6.

**TABLE 1:** Representative protein‐residue MMGBSA binding free‐energy contributions and ConSurf conservation grades at TSEN–pre‐tRNA interfaces.

Complex and Residue^a^, ^d^	Subunit^b^	Δ*G*_res_ ± SEM (kcal mol^−1^)	ConSurf grade^c^
AtTSEN–pre‐tRNA^Tyr^			
K61	AtTSEN54 (chain A)	−10.43 ± 0.09	5
R231	AtTSEN1 (chain C)	−5.59 ± 0.11	9
K6	AtTSEN1 (chain C)	−5.29 ± 0.10	9
R183	AtTSEN1 (chain C)	−4.93 ± 0.11	9
HsTSEN–pre‐tRNA^Arg^			
Y16	HsTSEN2	−10.70 ± 0.09	5
R13	HsTSEN2	−10.66 ± 0.12	9
R202	HsTSEN54	−9.47 ± 0.12	7
K97	HsTSEN54	−8.21 ± 0.10	9
R41	HsTSEN34	−8.19 ± 0.11	9
R171	HsTSEN15	−4.38 ± 0.12	9
ScTSEN–pre‐tRNA^Phe^			
R50	ScTSEN54	−8.60 ± 0.11	9
K458	ScTSEN54	−7.89 ± 0.10	1
R374	ScTSEN2	−7.15 ± 0.12	8
R376	ScTSEN2	−5.93 ± 0.12	9
R369	ScTSEN2	−5.82 ± 0.11	9

Abbreviations: Δ*G*_res_, per‐residue binding free energy; MMGBSA, molecular mechanics generalized Born surface area; pre‐tRNA, precursor transfer RNA; SEM, standard error of the mean.

^a^ Residues are denoted by single‐letter amino acid code followed by position number.

^b^ Chains given in parentheses to AtTSEN only, as two identical copies of each subunit are present; chain A and chain C denote the contributing protomers.

^c^ ConSurf conservation grade (1–3, variable; 4–6, intermediate; 7–9, highly conserved).

^d^ Organism prefixes: At, *Arabidopsis thaliana* ; Hs, *Homo sapiens* ; Sc, *Saccharomyces cerevisiae* .

Although RNA residues produced large decomposition energies owing to the polyanionic phosphate backbone, only protein residues are reported, as the present analysis focuses on protein determinants of pre‐tRNA recognition and their evolutionary conservation.

### 2.6 Evolutionary Conservation Analysis

Per‐residue evolutionary conservation was assessed using the ConSurf web server [54]. Each protein chain in the TSEN–pre‐tRNA AF3 complex models was submitted independently using the default ConSurf pipeline, with homolog retrieval against UniRef90 [55], a maximum of 150 hits, and a maximum pairwise sequence identity of 90%. The resulting ConSurf‐output PDB files, with conservation grades (1–9) stored in the B‐factor columns, were visualized in PyMOL using a modified ConSurf Python script to map conservation grades onto the complex structures. Conservation grades were integrated with MMGBSA decomposition values and reported for representative top‐ranked putative hotspot residues in Table 1, with the complete ranked list of protein residues meeting the Δ*G*_res_ ≤ −1 kcal mol^−1^ threshold provided in Table S6.

## 3 Results

### 3.1 AtTSEN54 Is Structurally More Similar to HsTSEN54 Than to ScTSEN54

A structure‐based homology search using the AF‐predicted ScTSEN54 model identified two putative AtTSEN54 homologs in the AlphaFold Database (AFDB): Q6DBN3 (254 aa; TM‐score = 0.62) and Q9FWA2 (201 aa; TM‐score = 0.55). These candidates share ~73% sequence similarity (Figure S1). Because Q6DBN3 showed the higher TM‐score and stronger AF3 confidence metrics, it was selected for subsequent AtTSEN modeling and comparative analyses.

For structural comparison, the AF‐predicted AtTSEN54 model was aligned with the AF‐predicted ScTSEN54 and cryo‐EM‐derived HsTSEN54 (PDB: 8HMY, chain C) [15] (Figure 1A,B). All‐atom superposition with HsTSEN54 revealed strong N‐terminal structural similarity, with the HsTSEN54 N‐terminal domain (NTD) aligning to AtTSEN54 at an RMSD of ~1.17 Å across 541 aligned atoms. By contrast, alignment with ScTSEN54 yielded an RMSD of ~23.2 Å across 1195 aligned atoms, indicating substantial structural divergence (Figure 1A,B).

**FIGURE 1:**
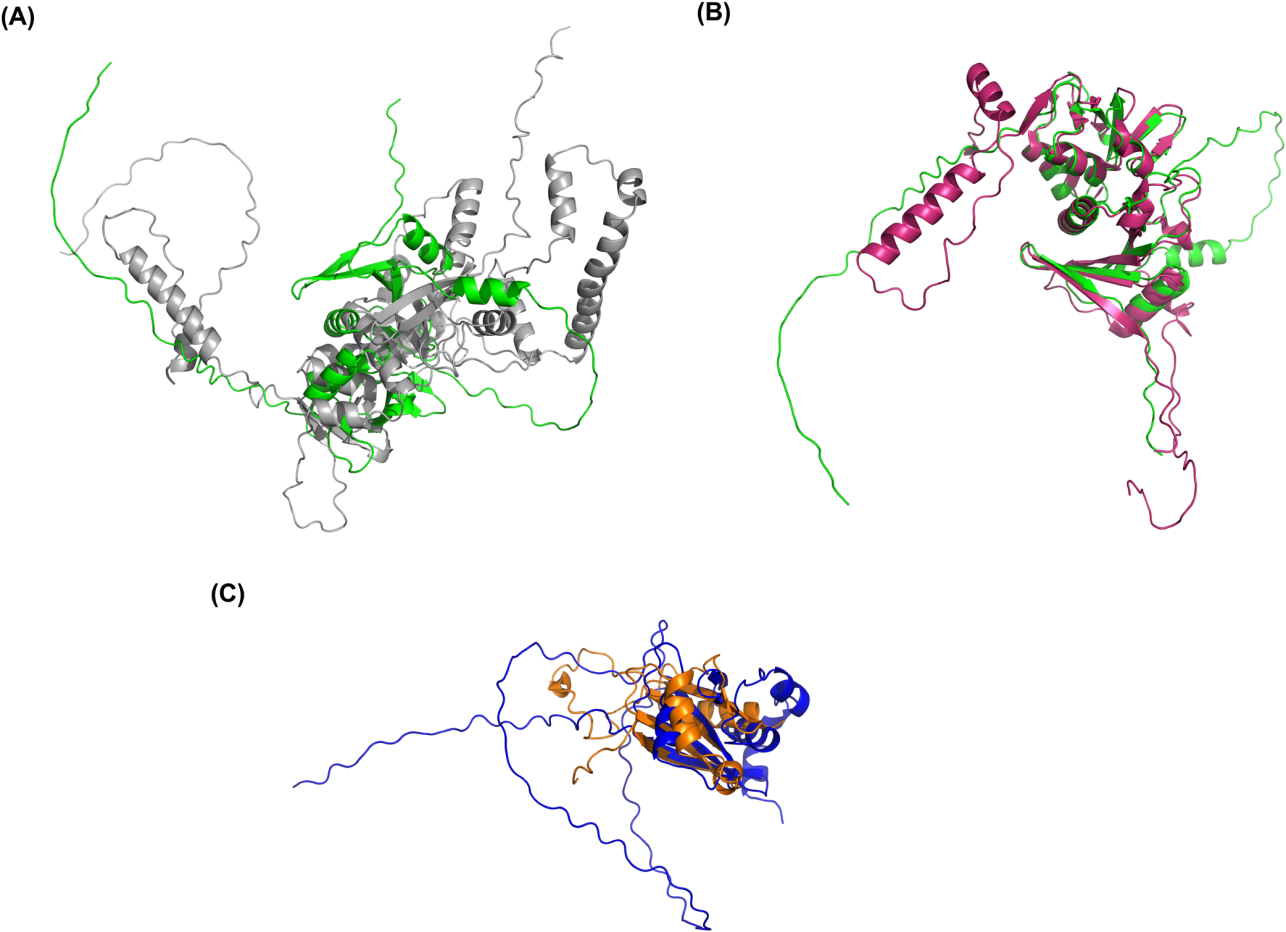
Structural superpositions of TSEN54 and TSEN15 homologs. (A) Superposition of AtTSEN54 with ScTSEN54; AtTSEN54 is shown in green and ScTSEN54 in gray. (B) Superposition of AtTSEN54 with HsTSEN54 (PDB: 8HMY, chain C); AtTSEN54 is shown in green and HsTSEN54 in warm pink. (C) Superposition of ScTSEN15 with CeTSEN15, a putative TSEN15 homolog from *Chlamydomonas eustigma*; ScTSEN15 is shown in orange and CeTSEN15 in marine blue. Organism prefixes: At, *Arabidopsis thaliana* ; Ce, *Chlamydomonas eustigma*; Hs, *Homo sapiens* ; Sc, *Saccharomyces cerevisiae* . Abbreviation: PDB, Protein Data Bank.

This structural similarity is not reflected at the sequence level. Pairwise sequence identity is low for both AtTSEN54–HsTSEN54 (23.1%) and AtTSEN54–ScTSEN54 (22.8%), calculated using Clustal Omega version 1.2.4 [56]. In both comparisons, sequence conservation is concentrated primarily within the N‐terminal region (Figure S2).

We next searched for a plant homolog of the second structural subunit, TSEN15. Sequence‐based searches using ScTSEN15 as the query did not identify convincing plant TSEN15 homologs. A complementary Foldseek search returned no high‐confidence homologs in *Arabidopsis* or other land plants. Notably, the best structural hit in the AFDB was an algal protein from *Chlamydomonas eustigma* (CeTSEN15; TM‐score = 0.45), which showed only partial structural similarity to ScTSEN15 (Figure 1C).

### 3.2 AtTSEN54 Interacts With Both AtTSEN1 and AtTSEN2 *in Planta*

To investigate potential protein–protein interactions among AtTSEN subunits, we performed bimolecular fluorescence complementation (BiFC) assays by transiently expressing N‐ and C‐terminal split‐Venus fusion constructs of AtTSEN1, AtTSEN2, and AtTSEN54 in onion epidermal cells. Co‐expression of AtTSEN54 with either AtTSEN1 or AtTSEN2 resulted in strong cytoplasmic Venus fluorescence under the conditions tested (Figure 2A,B). For the AtTSEN1–AtTSEN54 pair, four of the eight total combinations were positive; similarly, four of the eight were positive for the AtTSEN2–AtTSEN54 pair. In contrast, co‐expression of the catalytic subunits AtTSEN1 and AtTSEN2 did not yield any detectable fluorescence under identical conditions, suggesting no direct interaction between these two subunits (Figure S3). For both the AtTSEN1–AtTSEN54 and AtTSEN2–AtTSEN54 pairs, three of the eight BiFC combinations showed fluorescence signals significantly above the empty‐vector control (Figure 2C,D). Together, these findings support *in planta* interactions between AtTSEN54 and both AtTSEN1 and AtTSEN2.

**FIGURE 2:**
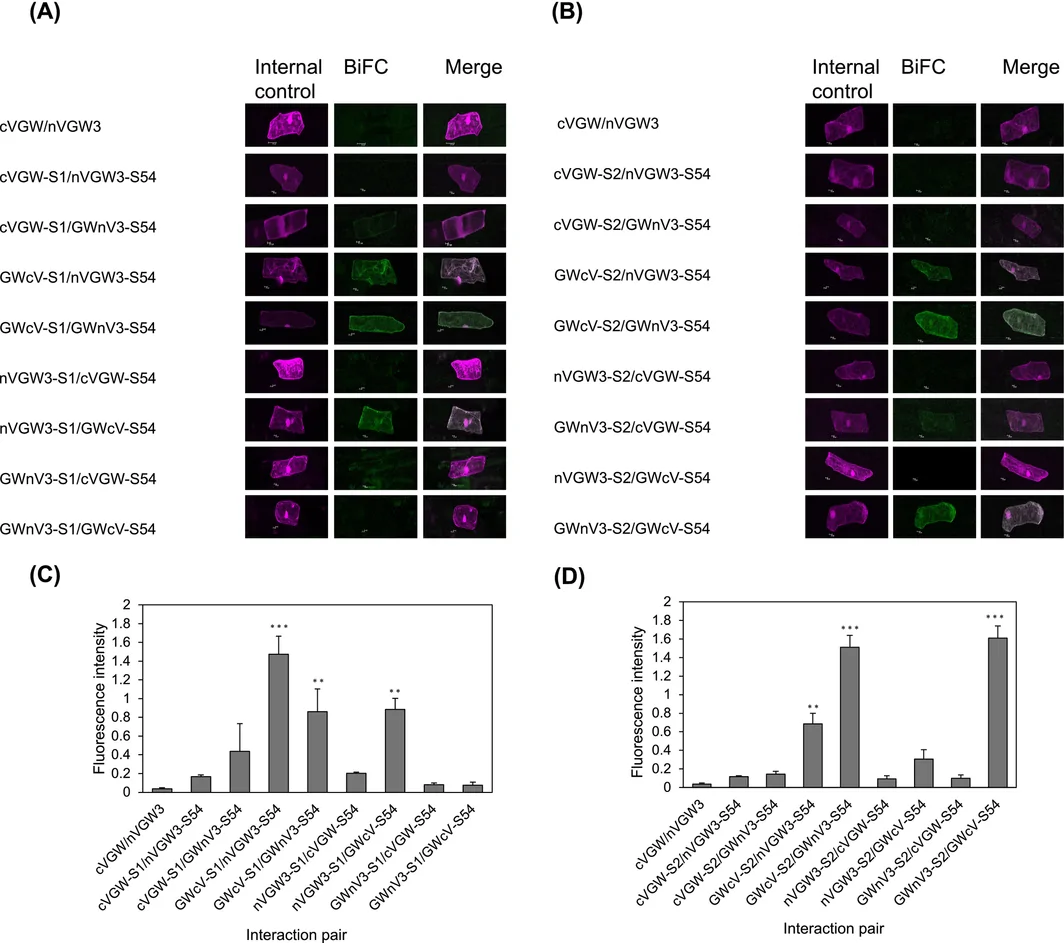
Bimolecular fluorescence complementation (BiFC) analysis of AtTSEN subunit interactions. (A, B) Representative confocal micrographs of onion epidermal cells showing reconstituted split‐Venus fluorescence for (A) AtTSEN1–AtTSEN54 and (B) AtTSEN2–AtTSEN54. For each panel, columns show the internal ECFP control channel (magenta), the BiFC Venus channel (green), and the merged overlay. Scale bars, 50 μm. A non‐interacting vector pair (pB4cVGW/pB4nVGW3) served as the negative control. (C, D) Quantification of BiFC signal intensity, expressed as the Venus/ECFP fluorescence ratio, for (C) AtTSEN1–AtTSEN54 and (D) AtTSEN2–AtTSEN54. Bars indicate mean ± SEM (*n* = 4 cells per condition). Experiments were performed using three biological replicates. Statistical comparisons were performed using one‐way analysis of variance (ANOVA) followed by Dunnett's post hoc test (*p ≤ 0.05; **p ≤ 0.01; ***p ≤ 0.001). In panel labels, vector names omit the pB4 prefix; protein names are abbreviated as S1 (AtTSEN1), S2 (AtTSEN2), and S54 (AtTSEN54). ANOVA, analysis of variance; ECFP, enhanced cyan fluorescent protein; μm, micrometer; SEM, standard error of the mean.

### 3.3 AF3 Predicts an (*αβ*)_2_‐Type AtTSEN Tetramer

AtTSEN1 and AtTSEN2 share ~72% sequence identity [17], and structural superposition of their AF models showed that this similarity extends to their three‐dimensional architecture, yielding an RMSD of ~0.34 Å over 1387 aligned atoms (Figure 3A). The AtTSEN1‐based tetramer model therefore serves as a structural proxy for an (*αβ*)_2_ AtTSEN architecture containing two catalytic *α*‐type subunits.

**FIGURE 3:**
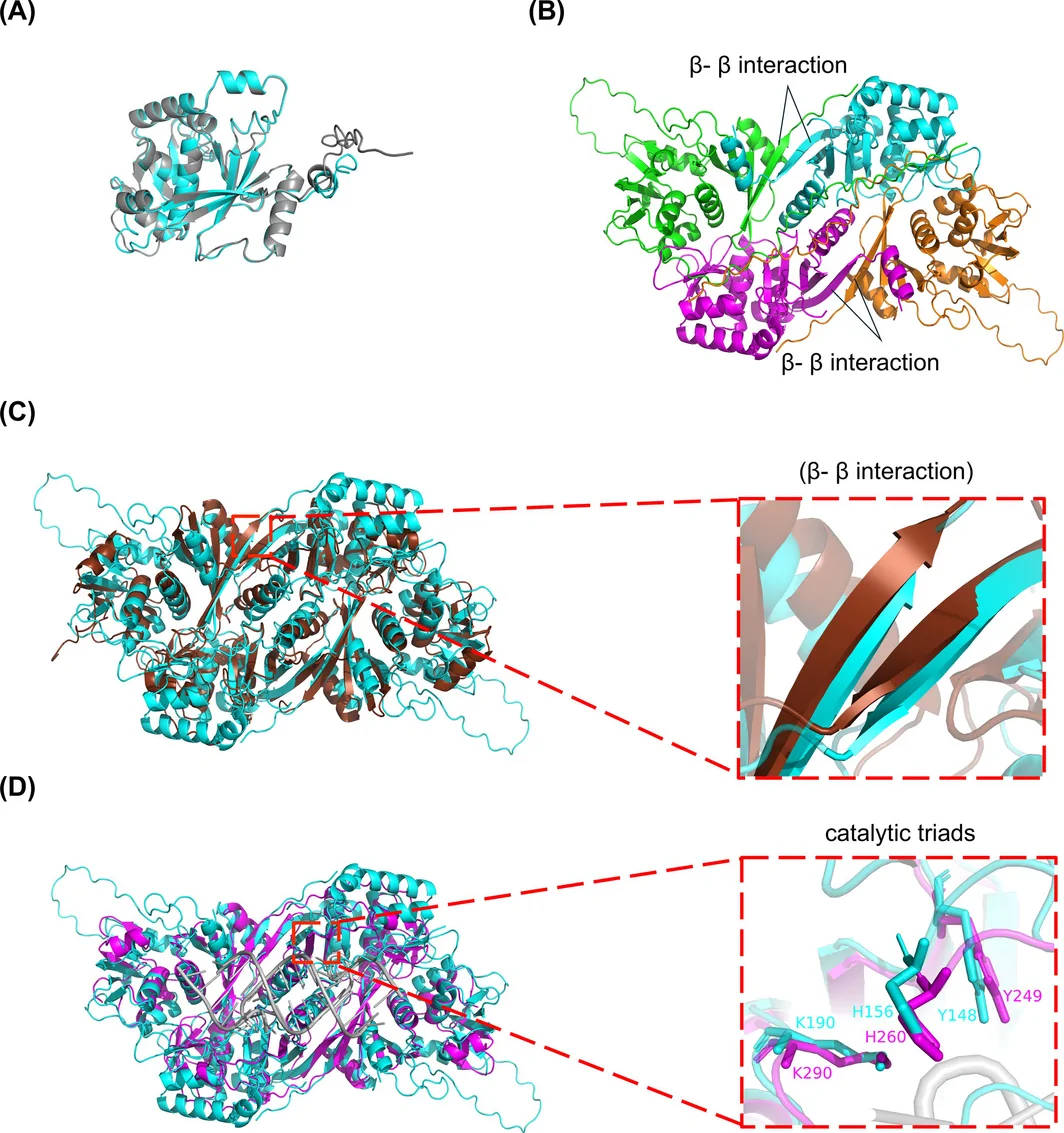
Structural similarity of AtTSEN catalytic subunits and comparison with archaeal TSEN complexes. (A) Superposition of AtTSEN1 (cyan) and AtTSEN2 (blue). (B) AlphaFold3 model of the AtTSEN complex. The two AtTSEN1 protomers are colored green (protomer 1) and orange (protomer 2), and the two AtTSEN54 protomers are colored cyan (protomer 1) and magenta (protomer 2). C‐terminal *β*–*β* interactions are indicated at each heterodimeric interface. (C) Superposition of AtTSEN (cyan) with *Aeropyrum pernix* TSEN (ApTSEN; chocolate; PDB: 3P1Z); left, overview with the active‐site region highlighted by a dashed box; right, close‐up of C‐terminal *β*–*β* interactions at the dimeric interface. (D) Superposition of AtTSEN (cyan) with *Archaeoglobus fulgidus* TSEN (AfTSEN; magenta; PDB: 2GJW) bound to bulge–helix–bulge (BHB) RNA (gray); left, overview with the active‐site region highlighted by a dashed box; right, close‐up of the catalytic triads labeled using one‐letter amino acid codes (AtTSEN: Y148, H156, and K190; AfTSEN: Y249, H260, and K290). Organism prefixes: Af, *Archaeoglobus fulgidus* ; Ap, *Aeropyrum pernix* ; At, *Arabidopsis thaliana* . BHB, bulge–helix–bulge.

Building on this structural equivalence, an AF3 tetramer was modeled comprising two catalytic *α*‐type protomers (AtTSEN1) and two structural *β*‐type protomers (AtTSEN54), in the absence of a detectable canonical TSEN15 homolog in *Arabidopsis*. The model revealed a pseudo‐symmetric (*αβ*)_2_ tetramer formed by antiparallel association of two structurally similar *αβ* heterodimers (Figure 3B; C*α* RMSD = 1.85 Å between heterodimers). Within the assembly, C‐terminal *β*‐strands of each *αβ* pair align in an antiparallel orientation, recapitulating the *β*–*β* interface conserved across archaeal and eukaryotic TSENs and implicated in stabilizing the multimeric assembly [4, 6].

The AtTSEN complex adopts a rectangular core architecture consistent with the archaeal (*αβ*)_2_ fold, as supported by superposition onto *Aeropyrum pernix* TSEN (PDB: 3P1Z; RMSD ~3.16 Å over 1627 atoms) [57] and *Archaeoglobus fulgidus* TSEN bound to BHB RNA (PDB: 2GJW; RMSD ~3.35 Å over 1581 atoms) [58], with side‐by‐side heterodimers and mirrored *β*–*β* interfaces (Figure 3C,D). Catalytic triad residues Y148, H156, and K190 overlay with the archaeal equivalents Y249, H260, and K290, supporting conservation of active‐site geometry (Figure 3D).

### 3.4 AF3 Modeling Suggests Conserved Pre‐tRNA Positioning and Electrostatic Complementarity

To investigate the structural basis of pre‐tRNA binding, we generated an AF3 model of the AtTSEN complex bound to pre‐tRNA^Tyr^ (Figure 4A). In this model, the L‐shaped pre‐tRNA occupies a continuous surface groove of the complex. One AtTSEN54 protomer is positioned against the acceptor stem and elbow region, consistent with the elbow‐binding role established for HsTSEN54 in the human cryo‐EM structure [6]. The anticodon arm, which harbors the intron loop, is oriented toward the catalytic centers. The two catalytic *α*‐type protomers occupy non‐equivalent substrate‐facing positions, with one adjacent to the 5′ splice site and the other adjacent to the 3′ splice site. This spatial arrangement corresponds to that observed in HsTSEN (Figure 4B), where HsTSEN2 and HsTSEN34 engage the 5′ and 3′ splice sites, respectively [6].

**FIGURE 4:**
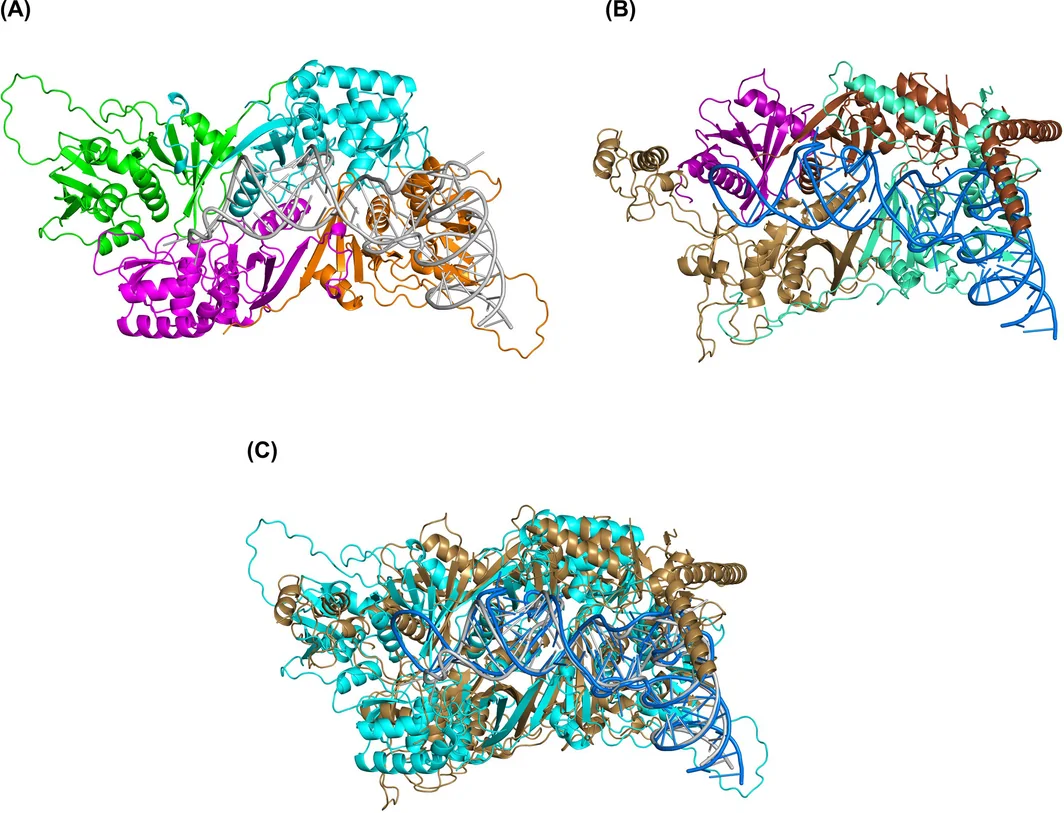
Conserved architecture of TSEN–pre‐tRNA complexes. (A) AlphaFold3 model of the AtTSEN–pre‐tRNA^Tyr^ complex. The two AtTSEN1 protomers are colored green (protomer 1) and orange (protomer 2), and the two AtTSEN54 protomers are colored cyan (protomer 1) and magenta (protomer 2); pre‐tRNA^Tyr^ is shown in gray. (B) Cryo‐electron microscopy (cryo‐EM) structure of the HsTSEN–pre‐tRNA^Arg^ complex (PDB: 8HMY). HsTSEN2 is shown in sand, HsTSEN34 in chocolate, HsTSEN54 in green‐cyan, HsTSEN15 in magenta, and pre‐tRNA^Arg^ in marine blue. (C) Superposition of the AtTSEN–pre‐tRNA^Tyr^ complex with the HsTSEN–pre‐tRNA^Arg^ complex (PDB: 8HMY). AtTSEN is shown in cyan, HsTSEN in sand, pre‐tRNA^Tyr^ in gray, and pre‐tRNA^Arg^ in marine blue. pre‐tRNA, precursor transfer RNA.

Superposition of the AtTSEN–pre‐tRNA^Tyr^ model onto the cryo‐EM structure of the HsTSEN–pre‐tRNA^Arg^ complex (PDB: 8HMY) [15], using the mature tRNA core for alignment, yielded an RNA RMSD of ~33.6 Å over 63 aligned atoms (Figure 4C). This high global RNA RMSD mainly reflects positional differences in the flexible intron‐containing anticodon arm. Despite this difference, the mature tRNA elbow and overall protein subunit arrangement remained comparable between the two complexes, with the AtTSEN1 protomers occupying positions corresponding to HsTSEN2 and HsTSEN34, and AtTSEN54 aligning with the elbow‐binding position of HsTSEN54.

ESP maps computed for the AtTSEN–pre‐tRNA^Tyr^, HsTSEN–pre‐tRNA^Arg^, and ScTSEN–pre‐tRNA^Phe^ complexes revealed positively charged surface grooves that accommodate the negatively charged RNA backbone (Figure 5A–C).

**FIGURE 5:**
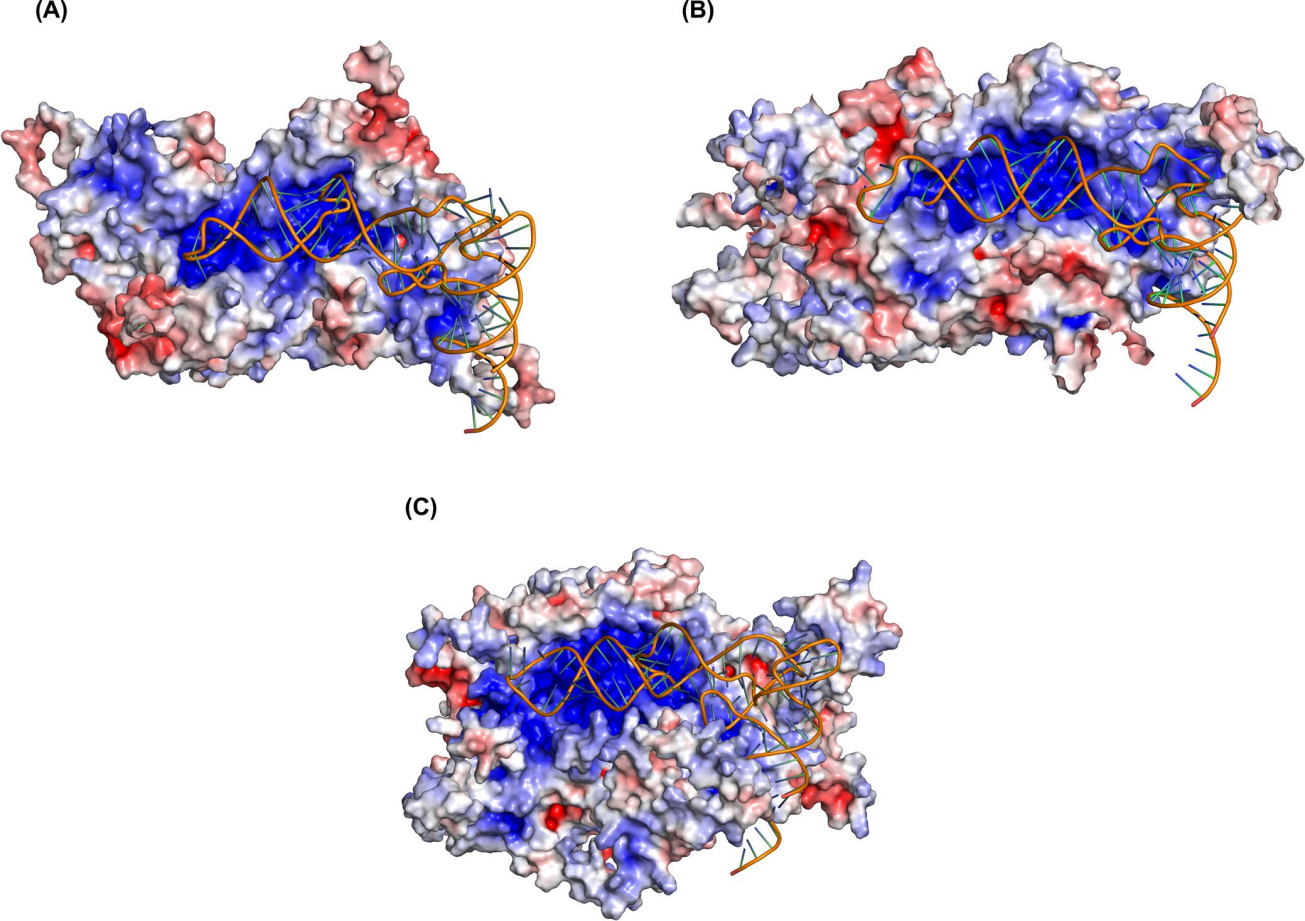
Electrostatic surface potential (ESP) analysis of TSEN–pre‐tRNA complexes. ESP was computed using the Adaptive Poisson–Boltzmann Solver (APBS) and mapped onto the solvent‐accessible surfaces of the protein components for (A) AtTSEN–pre‐tRNA^Tyr^, (B) HsTSEN–pre‐tRNA^Arg^, and (C) ScTSEN–pre‐tRNA^Phe^. The electrostatic potential scale ranges from −5 to +5 kT e^−1^, with negative potential shown in red and positive potential in blue. Pre‐tRNA molecules are shown as orange cartoon ribbons to indicate binding geometry only; RNA electrostatic potentials were not calculated.

### 3.5 Binding Free‐Energy Decomposition Reveals Basic Residues as Dominant Hotspot Contributors at the TSEN–Pre‐tRNA Interface

To identify putative hotspot residues at the protein–pre‐tRNA interface, we performed all‐atom MD simulations of the AF3‐predicted TSEN–pre‐tRNA complex models, followed by MMGBSA binding free‐energy decomposition over equilibrated trajectory intervals. RMSD analysis showed that all three complexes reached stable plateaus within the first 5–10 ns and remained relatively stable throughout the 100 ns simulations (Figure 6). AtTSEN–pre‐tRNA^Tyr^ stabilized at the lowest RMSD range (~0.25–0.32 nm), whereas HsTSEN–pre‐tRNA^Arg^ and ScTSEN–pre‐tRNA^Phe^ stabilized at ~0.50–0.55 nm, likely reflecting differences in complex size, retained flexible regions, and the extent of extended disordered segments.

**FIGURE 6:**
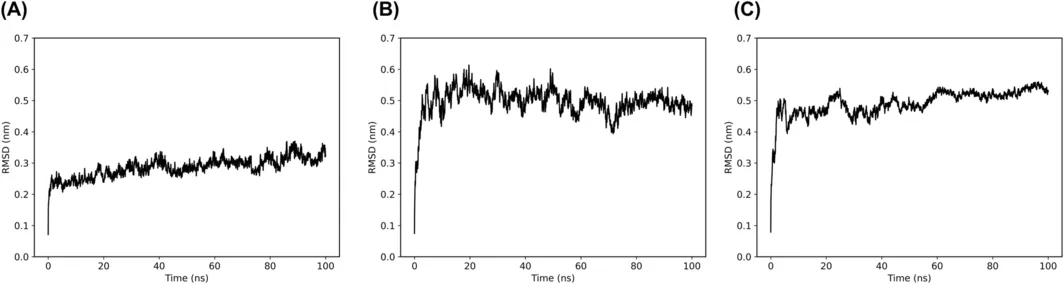
Backbone root‐mean‐square deviation (RMSD) profiles of TSEN–pre‐tRNA complexes during 100 ns molecular dynamics (MD) simulations. Backbone RMSD is shown as a function of simulation time for (A) AtTSEN–pre‐tRNA^Tyr^, (B) HsTSEN–pre‐tRNA^Arg^, and (C) ScTSEN–pre‐tRNA^Phe^. MD, molecular dynamics; nm, nanometers; ns, nanoseconds.

MMGBSA analysis of the equilibrated 70–100 ns interval yielded total binding free energies of −362.71 ± 0.30, −664.69 ± 0.36, and −728.83 ± 0.28 kcal mol^−1^ for the AtTSEN–pre‐tRNA^Tyr^, HsTSEN–pre‐tRNA^Arg^, and ScTSEN–pre‐tRNA^Phe^ complexes, respectively (Table S5). Because absolute MMGBSA values can scale with system size and interface composition, these values were interpreted comparatively rather than as direct estimates of experimental binding affinities.

Per‐residue MMGBSA decomposition showed that favorable energetic contributions clustered along the pre‐tRNA‐contacting surfaces in all three complexes, although the number and distribution of putative hotspot residues differed among orthologs (Figure 7A–C; Table S6). Across the three complexes, the strongest protein contributions were dominated by basic residues, particularly arginine and lysine, consistent with electrostatic stabilization of the RNA interface (Table 1). In AtTSEN, the two AtTSEN1 protomers collectively accounted for a large proportion of the top‐ranked residues, with R231, K6, and R183 positioned near the splice‐site regions. AtTSEN54 contributed through the predicted elbow‐contacting interface, where K61 was the strongest individual contributor. In HsTSEN, favorable contributions were concentrated mainly in HsTSEN2 and HsTSEN54: Y16 and R13 were among the strongest HsTSEN2 residues, R202 and K97 were prominent in HsTSEN54, and R41 was the leading HsTSEN34 residue, whereas HsTSEN15 showed only a minor direct contribution. In ScTSEN, ScTSEN54 was the dominant single‐subunit contributor, with R50 and K458 among the leading residues, followed by ScTSEN2 residues R374, R376, and R369. Representative top‐ranked protein hotspot residues are shown in Table 1, whereas the complete set of protein residues with individual Δ*G*_res_ ≤ −1 kcal mol^−1^, together with their ConSurf conservation grades, is provided in Table S6.

**FIGURE 7:**
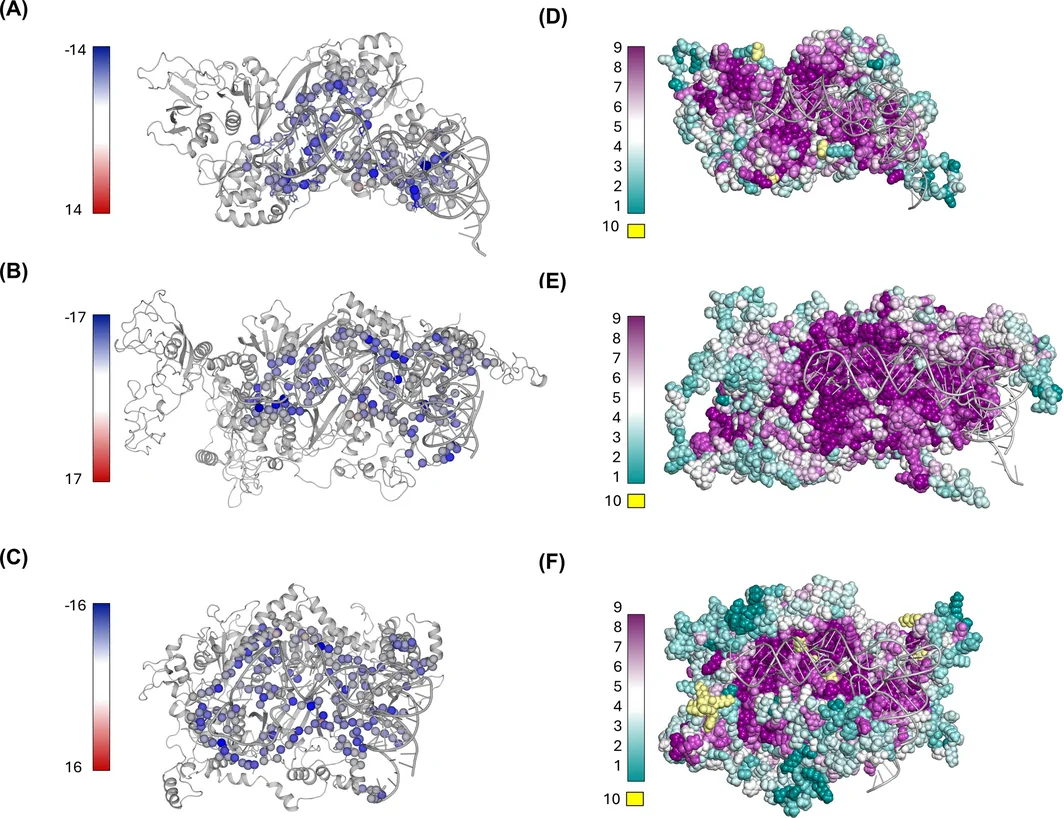
Per‐residue MMGBSA decomposition of binding free energy and ConSurf evolutionary conservation of TSEN–pre‐tRNA complexes. (A–C) Interface residues of AtTSEN–pre‐tRNA^Tyr^ (A), HsTSEN–pre‐tRNA^Arg^ (B), and ScTSEN–pre‐tRNA^Phe^ (C) shown as C*α* spheres colored by per‐residue MMGBSA binding free energy (kcal mol^−1^), with favorable contributions in blue and unfavorable contributions in red; energy scales are shown at left for each complex. (D–F) Corresponding structures shown as C*α* spheres colored by ConSurf conservation grade (grades 1–9; cyan–white–magenta gradient); scales are shown at right for each panel, where grades 6–9 indicate highly conserved, 4–5 intermediate, and 1–3 variable positions; yellow denotes insufficient data. MMGBSA decomposition was calculated for the protein–RNA interface; however, only protein residues are shown and reported because this analysis focused on protein‐side determinants of pre‐tRNA recognition and evolutionary conservation. Abbreviations: C*α*, alpha carbon; kcal mol^−1^, kilocalories per mole; MMGBSA, molecular mechanics generalized Born surface area.

ConSurf conservation mapping showed that core interface residues were generally conserved across AtTSEN, HsTSEN, and ScTSEN, whereas peripheral regions displayed greater variability (Figure 7D–F). TSEN15 made little contribution to the top‐ranked pre‐tRNA‐interacting residues in the eukaryotic reference complexes: no ScTSEN15 residue appeared among the leading contributors, and only one HsTSEN15 residue was identified among the favorable contributors (HsTSEN15‐R171; Table S6). This suggests that TSEN15 has a limited direct energetic role in pre‐tRNA binding in these models. Among the top energetic residues in each complex, most corresponded to ConSurf grades 7–9 (Tables 1 and S6), indicating that the principal pre‐tRNA‐binding interface is evolutionarily constrained, whereas peripheral contacts are species‐specific.

## 4 Discussion

Our BiFC analysis provided *in planta* evidence for subunit associations within the *Arabidopsis* TSEN complex. AtTSEN1 and AtTSEN2 each interacted with the structural subunit AtTSEN54, whereas no interaction was detected between AtTSEN1 and AtTSEN2 (Figures 2 and S3). This interaction pattern suggests that AtTSEN54 may scaffold catalytic *α*‐type subunits through heterodimeric contacts rather than through direct catalytic‐subunit pairing. This pairing logic is broadly consistent with the heterodimeric organization reported for ScTSEN and HsTSEN [3, 59]. Because BiFC reports proximity of fusion proteins in living plant cells, future co‐immunoprecipitation experiments using epitope‐tagged AtTSEN subunits will be important for independently corroborating these interactions and resolving intermediate assembly subcomplexes.

The structural analyses provide a plausible basis for this interaction pattern. AtTSEN1 and AtTSEN2 share ~72% sequence identity [17], and superposition of their AF3‐predicted structures revealed a high degree of similarity (Figure 3A). This similarity supports the use of an AtTSEN1‐based tetramer as a structural proxy for an AtTSEN assembly containing two catalytic *α*‐type protomers, while not implying that the native complex necessarily contains two identical AtTSEN1 copies. Consistent with partial functional overlap between the two catalytic subunits, genetic knockout studies indicate that AtTSEN2 can largely, but not fully, compensate for the loss of AtTSEN1, whereas reciprocal compensation by AtTSEN1 is weaker [60]. Superposition of AtTSEN54 with HsTSEN54 from the pre‐catalytic cryo‐EM structure (PDB: 8HMY) revealed strong conservation of the N‐terminal domain, which anchors the pre‐tRNA elbow in human TSEN [15] (Figure 1B). By contrast, AtTSEN54 lacks the extended C‐terminal region present in ScTSEN54 and HsTSEN54, suggesting lineage‐specific truncation or remodeling of peripheral structural elements while the core TSEN54 fold is retained. Superposition of the AtTSEN complex with the *A. pernix* and *A. fulgidus* TSEN structures showed a comparable rectangular arrangement, conserved *β*–*β* interfaces between catalytic and structural subunits, and preservation of the Tyr‐His‐Lys catalytic triad at each active site [57, 58] (Figure 3C,D). Together, these structural comparisons support an archaeal‐like (*αβ*)_2_ architecture for AtTSEN.

The apparent absence of a canonical TSEN15 homolog in land plants is consistent with this reduced‐subunit architectural model. Despite sequence‐ and structure‐based searches, we did not detect a convincing TSEN15 counterpart in *Arabidopsis* or other land plants. However, a candidate TSEN15‐like protein was identified in the green alga *C. eustigma* (Figure 1C), raising the possibility that TSEN15‐like proteins were retained in some algal lineages but lost, replaced, or highly diverged in land plants. Because TSEN15 is small and poorly conserved, non‐detection by homology searches does not exclude the existence of a highly divergent accessory factor. Thus, the AtTSEN model should be viewed as a testable structural hypothesis rather than a definitive inventory of all plant TSEN‐associated proteins.

The pre‐tRNA‐bound AF3 model links this proposed architecture to substrate recognition. Superposition of the AtTSEN–pre‐tRNA^Tyr^ model onto the HsTSEN–pre‐tRNA^Arg^ cryo‐EM complex (PDB: 8HMY) [15] showed a broadly similar arrangement, with the mature tRNA body positioned along the AtTSEN catalytic groove in a manner analogous to the human complex [6, 15, 16] (Figure 4C). In this model, AtTSEN54 occupies the elbow‐contacting position, whereas the two catalytic *α*‐type protomers lie near the predicted 5′ and 3′ splice‐site regions. Thus, the protein scaffold is pseudo‐symmetric, but pre‐tRNA binding imposes functional asymmetry at the catalytic interface. ESP mapping further showed an extended electropositive groove compatible with the negatively charged pre‐tRNA backbone in the AtTSEN–pre‐tRNA^Tyr^ model (Figure 5A). Comparable electropositive groove patterns were observed in the HsTSEN–pre‐tRNA^Arg^ and ScTSEN–pre‐tRNA^Phe^ models (Figure 5B,C), with the HsTSEN pattern consistent with reported human TSEN–pre‐tRNA cryo‐EM structures [15, 16]. These shared features support electrostatic complementarity as a conserved mode of TSEN substrate engagement and suggest that AtTSEN may preserve core RNA‐positioning principles while using a remodeled plant‐specific subunit arrangement.

MMGBSA decomposition and ConSurf mapping further support this view by identifying energetically favorable and evolutionarily constrained residues at the predicted TSEN–pre‐tRNA interface. Across the AtTSEN, HsTSEN, and ScTSEN models, favorable contributions were dominated by basic protein residues, particularly Arg and Lys, consistent with electrostatic stabilization of the RNA backbone. In AtTSEN, several strong contributors were located in AtTSEN1 near the predicted splice‐site regions, whereas AtTSEN54 contributed at the predicted elbow‐contacting interface. Comparable energetic hotspots were also observed in the human and yeast reference complexes, although their subunit distribution differed among lineages. ConSurf analysis showed that many of the strongest MMGBSA contributors corresponded to highly conserved positions, whereas peripheral interface contacts were more variable (Figure 7D–F; Tables 1 and S6). This overlap between energetic contribution and evolutionary conservation suggests that a conserved set of basic interface residues helps stabilize pre‐tRNA recognition, while lineage‐specific residues may tune substrate engagement in different TSEN architectures. These putative hotspots provide priority candidates for structure‐guided mutagenesis to test their predicted roles in elbow engagement, splice‐site positioning, and catalytic activity.

Taken together, our results support an archaeal‐like (*αβ*)_2_ heterotetrameric model for AtTSEN, organized as two structurally similar *αβ* heterodimers that are positioned in functionally non‐equivalent orientations in the pre‐tRNA‐bound state. The BHL‐type architecture of the modeled plant pre‐tRNA substrate positions the splice sites asymmetrically relative to the anticodon. Given this substrate context, the reported inability of plant tRNA endonucleases to cleave heterologous intron‐containing substrates further supports enzyme–substrate co‐adaptation [24]. Whether AtTSEN can process BHB‐containing substrates, as observed for archaeal (*αβ*)_2_ endonucleases [4, 10, 11], remains an experimentally testable question. A relevant precedent is provided by the red alga *Cyanidioschyzon merolae*, in which tRNA splicing is mediated by TSEN2‐, TSEN34‐, and TSEN54‐like proteins despite circularly permuted pre‐tRNA architectures processed through BHB‐like intermediates [61, 62]. This observation suggests that reduced‐subunit eukaryotic TSEN systems can accommodate diverse pre‐tRNA configurations. Whether a plant‐specific accessory factor analogous to TSEN15 exists also remains unresolved, especially because TSEN15 in yeast and humans was identified through biochemical purification rather than sequence similarity searches [3, 5]. Targeted affinity purification of AtTSEN subunits from *Arabidopsis* extracts, together with in vitro splicing assays using reconstituted complexes, will therefore be essential for testing subunit composition and catalytic sufficiency. Ultimately, cryo‐EM analysis of the native AtTSEN–pre‐tRNA complex would provide direct experimental validation of the proposed architecture and clarify how plant‐specific remodeling contributed to the evolutionary diversification of TSEN complexes.

## Author Contributions

**Kazuhito Akama:** investigation, conceptualization, supervision, writing – review and editing. **Mohammad Moniruzzaman:** investigation, writing – original draft, conceptualization, methodology, visualization, formal analysis, writing – review and editing. **Markus Englert:** investigation, conceptualization, methodology, visualization, writing – original draft, formal analysis.

## Funding

This work was supported by Japan Science and Technology Agency (JPMJSP2155).

## Conflicts of Interest

The authors declare no conflicts of interest.

## Supporting information


**Figure S1:** Multiple sequence alignment of putative AtTSEN54 homologs from *Arabidopsis thaliana* .
**Figure S2:** Multiple sequence alignment of TSEN54 homologs from *Arabidopsis thaliana* , *Homo sapiens* , and *Saccharomyces cerevisiae* .
**Figure S3:** Bimolecular fluorescence complementation (BiFC) analysis of AtTSEN1–AtTSEN2 interactions.
**Figure S4:** Confidence assessment of AlphaFold3 (AF3) TSEN complex models generated in this study.

**Table S1:** Database identifiers of protein monomers and pre‐tRNA sequences used in this study.

**Table S2:** Primers used for cloning in this study

**Table S3:** Plasmids used for bimolecular fluorescence complementation analysis (BiFC).

**Table S4:** BiFC construct combinations used in this study.

**Table S5:** MMGBSA binding free‐energy components for TSEN–pre‐tRNA complexes.

**Table S6:** Protein‐side per‐residue MMGBSA binding free‐energy contributions and ConSurf conservation grades at TSEN–pre‐tRNA interfaces.

## Data Availability

The data that support the findings of this study are available on request from the corresponding author. The data are not publicly available due to privacy or ethical restrictions.
