# Supplementary material for: An (αβ)2 Architecture of the Arabidopsis thaliana tRNA Splicing Endonuclease Complex: Structural Modeling and Evolutionary Analyses
Source: Proteins. 2026 Jul 9;94(12):1913–26. doi: 10.1002/prot.70155 (PMC13728533; doi:10.1002/prot.70155)
Supplement: Supplementary file 1 [file PROT-94-1913-s001.docx]

**Table S4.** BiFC construct combinations used in this study.

| **Protein pair** | **nVenus fusion construct** | **cVenus fusion construct** |
| --- | --- | --- |
| **AtTSEN1–AtTSEN54** |  |  |
|  | pB4GWnV3-AtTSEN1 | pB4cVGW-AtTSEN54 |
|  | pB4GWnV3-AtTSEN1 | pB4GWcV-AtTSEN54 |
|  | pB4nVGW3-AtTSEN1 | pB4cVGW-AtTSEN54 |
|  | pB4nVGW3-AtTSEN1 | pB4GWcV-AtTSEN54 |
|  | pB4GWnV3-AtTSEN54 | pB4cVGW-AtTSEN1 |
|  | pB4GWnV3-AtTSEN54 | pB4GWcV-AtTSEN1 |
|  | pB4nVGW3-AtTSEN54 | pB4cVGW-AtTSEN1 |
|  | pB4nVGW3-AtTSEN54 | pB4GWcV-AtTSEN1 |
| **AtTSEN1–AtTSEN2** |  |  |
|  | pB4GWnV3-AtTSEN1 | pB4cVGW-AtTSEN2 |
|  | pB4GWnV3-AtTSEN1 | pB4GWcV-AtTSEN2 |
|  | pB4nVGW3-AtTSEN1 | pB4cVGW-AtTSEN2 |
|  | pB4nVGW3-AtTSEN1 | pB4GWcV-AtTSEN2 |
|  | pB4GWnV3-AtTSEN2 | pB4cVGW-AtTSEN1 |
|  | pB4GWnV3-AtTSEN2 | pB4GWcV-AtTSEN1 |
|  | pB4nVGW3-AtTSEN2 | pB4cVGW-AtTSEN1 |
|  | pB4nVGW3-AtTSEN2 | pB4GWcV-AtTSEN1 |
| **AtTSEN2–AtTSEN54** |  |  |
|  | pB4GWnV3-AtTSEN2 | pB4cVGW-AtTSEN54 |
|  | pB4GWnV3-AtTSEN2 | pB4GWcV-AtTSEN54 |
|  | pB4nVGW3-AtTSEN2 | pB4cVGW-AtTSEN54 |
|  | pB4nVGW3-AtTSEN2 | pB4GWcV-AtTSEN54 |
|  | pB4GWnV3-AtTSEN54 | pB4cVGW-AtTSEN2 |
|  | pB4GWnV3-AtTSEN54 | pB4GWcV-AtTSEN2 |
|  | pB4nVGW3-AtTSEN54 | pB4cVGW-AtTSEN2 |
|  | pB4nVGW3-AtTSEN54 | pB4GWcV-AtTSEN2 |

Notes: nVenus, N-terminal fragment of Venus fluorescent protein; cVenus, C-terminal fragment of Venus fluorescent protein.
