# Supplementary material for: An (αβ)2 Architecture of the Arabidopsis thaliana tRNA Splicing Endonuclease Complex: Structural Modeling and Evolutionary Analyses
Source: Proteins. 2026 Jul 9;94(12):1913–26. doi: 10.1002/prot.70155 (PMC13728533; doi:10.1002/prot.70155)
Supplement: Supplementary file 2 [file PROT-94-1913-s002.docx]

Table S6. Protein-side per-residue MMGBSA binding free-energy contributions and ConSurf conservation grades at TSEN–pre-tRNA interfaces.

| **Complex and residue^†^** | **Subunit^‡^** | **ΔG_res_ ± SEM (kcal mol^−1^)** | | **ConSurf grade^§^** | |
| --- | --- | --- | --- | --- | --- |
| **AtTSEN–pre-tRNA^Tyr^** | | | | | |
| K61 | AtTSEN54 (chain A) | | −10.43 ± 0.09 | | 5 |
| R193 | AtTSEN54 (chain A) | | −6.18 ± 0.11 | | 7 |
| R59 | AtTSEN54 (chain A) | | −6.01 ± 0.12 | | 7 |
| K195 | AtTSEN54 (chain A) | | −5.23 ± 0.10 | | 6 |
| R239 | AtTSEN54 (chain A) | | −5.06 ± 0.12 | | 7 |
| K58 | AtTSEN54 (chain A) | | −4.56 ± 0.10 | | 5 |
| Q237 | AtTSEN54 (chain A) | | −3.90 ± 0.09 | | 5 |
| S192 | AtTSEN54 (chain A) | | −3.10 ± 0.06 | | 8 |
| K196 | AtTSEN54 (chain A) | | −3.09 ± 0.09 | | 9 |
| G60 | AtTSEN54 (chain A) | | −2.91 ± 0.05 | | 9 |
| L62 | AtTSEN54 (chain A) | | −2.88 ± 0.09 | | 4 |
| K145 | AtTSEN54 (chain A) | | −2.73 ± 0.11 | | 9 |
| I69 | AtTSEN54 (chain A) | | −2.52 ± 0.09 | | 4 |
| G40 | AtTSEN54 (chain A) | | −2.22 ± 0.12 | | 4 |
| T64 | AtTSEN54 (chain A) | | −2.18 ± 0.07 | | 5 |
| P190 | AtTSEN54 (chain A) | | −2.06 ± 0.07 | | 9 |
| L131 | AtTSEN54 (chain A) | | −1.97 ± 0.08 | | 9 |
| G72 | AtTSEN54 (chain A) | | −1.87 ± 0.05 | | 6 |
| N191 | AtTSEN54 (chain A) | | −1.85 ± 0.08 | | 9 |
| F194 | AtTSEN54 (chain A) | | −1.17 ± 0.08 | | 9 |
| G132 | AtTSEN54 (chain A) | | −1.00 ± 0.05 | | 9 |
| R239 | AtTSEN54 (chain C) | | −2.54 ± 0.12 | | 7 |
| R231 | AtTSEN1 (chain A) | | −5.59 ± 0.11 | | 9 |
| K6 | AtTSEN1 (chain A) | | −5.29 ± 0.10 | | 9 |
| R183 | AtTSEN1 (chain A) | | −4.93 ± 0.11 | | 9 |
| R4 | AtTSEN1 (chain A) | | −4.75 ± 0.12 | | 9 |
| V188 | AtTSEN1 (chain A) | | −4.12 ± 0.08 | | 9 |
| R224 | AtTSEN1 (chain A) | | −4.07 ± 0.12 | | 9 |
| H156 | AtTSEN1 (chain A) | | −4.03 ± 0.08 | | 9 |
| S187 | AtTSEN1 (chain A) | | −3.60 ± 0.07 | | 7 |
| K8 | AtTSEN1 (chain A) | | −2.61 ± 0.11 | | 7 |
| L229 | AtTSEN1 (chain A) | | −2.02 ± 0.09 | | 7 |
| Y141 | AtTSEN1 (chain A) | | −1.94 ± 0.09 | | 8 |
| W7 | AtTSEN1 (chain A) | | −1.92 ± 0.09 | | 7 |
| A189 | AtTSEN1 (chain A) | | −1.92 ± 0.06 | | 9 |
| R63 | AtTSEN1 (chain A) | | −1.91 ± 0.12 | | 4 |
| E228 | AtTSEN1 (chain A) | | −1.49 ± 0.12 | | 8 |
| R136 | AtTSEN1 (chain A) | | −1.29 ± 0.11 | | 7 |
| M1 | AtTSEN1 (chain A) | | −1.27 ± 0.12 | | 9 |
| S153 | AtTSEN1 (chain A) | | −1.23 ± 0.06 | | 7 |
| K190 | AtTSEN1 (chain A) | | −1.13 ± 0.10 | | 9 |
| T235 | AtTSEN1 (chain A) | | −1.13 ± 0.08 | | 5 |
| H179 | AtTSEN1 (chain A) | | −1.11 ± 0.08 | | 5 |
| W5 | AtTSEN1 (chain A) | | −1.06 ± 0.09 | | 8 |
| K6 | AtTSEN1 (chain C) | | −4.12 ± 0.10 | | 9 |
| R136 | AtTSEN1 (chain C) | | −3.99 ± 0.11 | | 7 |
| Q140 | AtTSEN1 (chain C) | | −3.71 ± 0.09 | | 7 |
| H156 | AtTSEN1 (chain C) | | −2.78 ± 0.08 | | 9 |
| S187 | AtTSEN1 (chain C) | | −2.69 ± 0.06 | | 7 |
| M1 | AtTSEN1 (chain C) | | −2.62 ± 0.10 | | 9 |
| R183 | AtTSEN1 (chain C) | | −2.45 ± 0.12 | | 9 |
| R63 | AtTSEN1 (chain C) | | −2.34 ± 0.12 | | 4 |
| P152 | AtTSEN1 (chain C) | | −1.65 ± 0.07 | | 9 |
| A2 | AtTSEN1 (chain C) | | −1.65 ± 0.06 | | 4 |
| R224 | AtTSEN1 (chain C) | | −1.40 ± 0.11 | | 9 |
| P3 | AtTSEN1 (chain C) | | −1.34 ± 0.07 | | 7 |
| L139 | AtTSEN1 (chain C) | | −1.29 ± 0.09 | | 5 |
| V188 | AtTSEN1 (chain C) | | −1.28 ± 0.08 | | 9 |
| K190 | AtTSEN1 (chain C) | | −1.03 ± 0.10 | | 9 |
| **HsTSEN–pre-tRNA^Arg^** | | | | | |
| R41 | HsTSEN34 | | −8.19 ± 0.11 | | 9 |
| R285 | HsTSEN34 | | −5.54 ± 0.12 | | 8 |
| I116 | HsTSEN34 | | −4.30 ± 0.09 | | 8 |
| R108 | HsTSEN34 | | −4.29 ± 0.14 | | 6 |
| R105 | HsTSEN34 | | −3.95 ± 0.12 | | 8 |
| R31 | HsTSEN34 | | −3.93 ± 0.11 | | 6 |
| Q120 | HsTSEN34 | | −3.11 ± 0.09 | | 7 |
| Q310 | HsTSEN34 | | −3.09 ± 0.11 | | 9 |
| R45 | HsTSEN34 | | −3.04 ± 0.11 | | 7 |
| R109 | HsTSEN34 | | −2.92 ± 0.13 | | 7 |
| R279 | HsTSEN34 | | −2.91 ± 0.11 | | 9 |
| P171 | HsTSEN34 | | −2.82 ± 0.08 | | 2 |
| K123 | HsTSEN34 | | −2.81 ± 0.14 | | 7 |
| Q42 | HsTSEN34 | | −2.57 ± 0.09 | | 9 |
| L112 | HsTSEN34 | | −2.10 ± 0.09 | | 6 |
| T282 | HsTSEN34 | | −2.06 ± 0.07 | | 8 |
| K239 | HsTSEN34 | | −1.92 ± 0.10 | | 9 |
| K126 | HsTSEN34 | | −1.75 ± 0.14 | | 4 |
| H255 | HsTSEN34 | | −1.67 ± 0.08 | | 9 |
| S44 | HsTSEN34 | | −1.66 ± 0.06 | | 4 |
| S283 | HsTSEN34 | | −1.65 ± 0.06 | | 9 |
| G119 | HsTSEN34 | | −1.58 ± 0.05 | | 8 |
| V284 | HsTSEN34 | | −1.54 ± 0.08 | | 9 |
| P251 | HsTSEN34 | | −1.18 ± 0.07 | | 9 |
| R38 | HsTSEN34 | | −1.14 ± 0.11 | | 9 |
| K286 | HsTSEN34 | | −1.13 ± 0.10 | | 9 |
| G170 | HsTSEN34 | | −1.10 ± 0.05 | | 4 |
| K115 | HsTSEN34 | | −1.05 ± 0.11 | | 5 |
| R171 | HsTSEN15 | | −4.38 ± 0.12 | | 9 |
| V151 | HsTSEN15 | | −2.40 ± 0.08 | | 9 |
| R170 | HsTSEN15 | | −2.09 ± 0.13 | | 8 |
| Y152 | HsTSEN15 | | −1.39 ± 0.08 | | 9 |
| Y16 | HsTSEN2 | | −10.70 ± 0.09 | | 5 |
| R13 | HsTSEN2 | | −10.66 ± 0.12 | | 9 |
| R14 | HsTSEN2 | | −8.14 ± 0.12 | | 9 |
| R459 | HsTSEN2 | | −5.59 ± 0.11 | | 9 |
| R73 | HsTSEN2 | | −5.18 ± 0.11 | | 9 |
| R75 | HsTSEN2 | | −4.44 ± 0.12 | | 9 |
| R457 | HsTSEN2 | | −4.42 ± 0.15 | | 9 |
| R11 | HsTSEN2 | | −4.40 ± 0.12 | | 9 |
| N413 | HsTSEN2 | | −4.34 ± 0.08 | | 8 |
| R409 | HsTSEN2 | | −4.27 ± 0.11 | | 9 |
| K12 | HsTSEN2 | | −4.09 ± 0.10 | | 9 |
| S74 | HsTSEN2 | | −4.04 ± 0.06 | | 9 |
| K10 | HsTSEN2 | | −3.94 ± 0.10 | | 7 |
| K361 | HsTSEN2 | | −3.93 ± 0.10 | | 9 |
| V412 | HsTSEN2 | | −2.60 ± 0.08 | | 5 |
| Q462 | HsTSEN2 | | −2.54 ± 0.09 | | 8 |
| P373 | HsTSEN2 | | −2.38 ± 0.07 | | 9 |
| R452 | HsTSEN2 | | −2.21 ± 0.11 | | 9 |
| V414 | HsTSEN2 | | −2.13 ± 0.08 | | 9 |
| K68 | HsTSEN2 | | −2.02 ± 0.09 | | 9 |
| E17 | HsTSEN2 | | −1.99 ± 0.08 | | 9 |
| S456 | HsTSEN2 | | −1.81 ± 0.06 | | 9 |
| P374 | HsTSEN2 | | −1.71 ± 0.07 | | 8 |
| S415 | HsTSEN2 | | −1.60 ± 0.07 | | 9 |
| K416 | HsTSEN2 | | −1.35 ± 0.10 | | 9 |
| H377 | HsTSEN2 | | −1.33 ± 0.08 | | 9 |
| K357 | HsTSEN2 | | −1.32 ± 0.09 | | 9 |
| V15 | HsTSEN2 | | −1.25 ± 0.07 | | 9 |
| S460 | HsTSEN2 | | −1.23 ± 0.07 | | 8 |
| R202 | HsTSEN54 | | −9.47 ± 0.12 | | 7 |
| K97 | HsTSEN54 | | −8.21 ± 0.10 | | 9 |
| R36 | HsTSEN54 | | −7.46 ± 0.11 | | 8 |
| K32 | HsTSEN54 | | −7.21 ± 0.10 | | 6 |
| R200 | HsTSEN54 | | −6.48 ± 0.11 | | 9 |
| Q31 | HsTSEN54 | | −6.30 ± 0.08 | | 6 |
| Q107 | HsTSEN54 | | −5.56 ± 0.10 | | 3 |
| R73 | HsTSEN54 | | −5.03 ± 0.12 | | 9 |
| R109 | HsTSEN54 | | −4.88 ± 0.12 | | 7 |
| R462 | HsTSEN54 | | −4.35 ± 0.13 | | 8 |
| Q100 | HsTSEN54 | | −3.94 ± 0.09 | | 8 |
| K201 | HsTSEN54 | | −3.65 ± 0.12 | | 8 |
| K41 | HsTSEN54 | | −3.42 ± 0.09 | | 9 |
| S203 | HsTSEN54 | | −3.33 ± 0.06 | | 8 |
| Q35 | HsTSEN54 | | −3.19 ± 0.09 | | 6 |
| G108 | HsTSEN54 | | −3.14 ± 0.05 | | 9 |
| R27 | HsTSEN54 | | −3.07 ± 0.12 | | 8 |
| W99 | HsTSEN54 | | −2.78 ± 0.09 | | 9 |
| P94 | HsTSEN54 | | −2.78 ± 0.07 | | 8 |
| K199 | HsTSEN54 | | −2.74 ± 0.10 | | 9 |
| L33 | HsTSEN54 | | −2.67 ± 0.09 | | 6 |
| R208 | HsTSEN54 | | −2.65 ± 0.13 | | 3 |
| K212 | HsTSEN54 | | −2.58 ± 0.13 | | 5 |
| K463 | HsTSEN54 | | −2.33 ± 0.09 | | 9 |
| R83 | HsTSEN54 | | −2.26 ± 0.12 | | 6 |
| G96 | HsTSEN54 | | −1.78 ± 0.05 | | 9 |
| P40 | HsTSEN54 | | −1.71 ± 0.07 | | 8 |
| S105 | HsTSEN54 | | −1.69 ± 0.06 | | 7 |
| A455 | HsTSEN54 | | −1.64 ± 0.06 | | 9 |
| G165 | HsTSEN54 | | −1.50 ± 0.05 | | 8 |
| R29 | HsTSEN54 | | −1.40 ± 0.13 | | 7 |
| S37 | HsTSEN54 | | −1.38 ± 0.07 | | 6 |
| R111 | HsTSEN54 | | −1.30 ± 0.12 | | 4 |
| K162 | HsTSEN54 | | −1.14 ± 0.10 | | 8 |
| Q110 | HsTSEN54 | | −1.06 ± 0.08 | | 9 |
| V458 | HsTSEN54 | | −1.00 ± 0.08 | | 8 |
| **ScTSEN–pre-tRNA^Phe^** | | | | | |
| R374 | ScTSEN2 | | −7.15 ± 0.12 | | 8 |
| R376 | ScTSEN2 | | −5.93 ± 0.12 | | 9 |
| R369 | ScTSEN2 | | −5.82 ± 0.11 | | 9 |
| L124 | ScTSEN2 | | −5.43 ± 0.08 | | 7 |
| K327 | ScTSEN2 | | −4.91 ± 0.10 | | 8 |
| R135 | ScTSEN2 | | −4.82 ± 0.11 | | 9 |
| R170 | ScTSEN2 | | −4.59 ± 0.11 | | 7 |
| K3 | ScTSEN2 | | −4.56 ± 0.10 | | 8 |
| R5 | ScTSEN2 | | −4.55 ± 0.12 | | 4 |
| R174 | ScTSEN2 | | −3.84 ± 0.12 | | 5 |
| R131 | ScTSEN2 | | −3.82 ± 0.12 | | 9 |
| R321 | ScTSEN2 | | −3.56 ± 0.12 | | 9 |
| K139 | ScTSEN2 | | −3.45 ± 0.09 | | 9 |
| H297 | ScTSEN2 | | −3.38 ± 0.08 | | 9 |
| R142 | ScTSEN2 | | −3.17 ± 0.11 | | 9 |
| Q8 | ScTSEN2 | | −3.14 ± 0.09 | | 6 |
| G4 | ScTSEN2 | | −3.13 ± 0.05 | | 5 |
| R88 | ScTSEN2 | | −2.70 ± 0.11 | | 9 |
| R132 | ScTSEN2 | | −2.41 ± 0.11 | | 9 |
| V371 | ScTSEN2 | | −2.33 ± 0.08 | | 6 |
| F295 | ScTSEN2 | | −2.27 ± 0.08 | | 9 |
| K177 | ScTSEN2 | | −1.91 ± 0.10 | | 7 |
| A326 | ScTSEN2 | | −1.82 ± 0.06 | | 9 |
| G373 | ScTSEN2 | | −1.80 ± 0.05 | | 8 |
| F282 | ScTSEN2 | | −1.71 ± 0.09 | | 9 |
| S2 | ScTSEN2 | | −1.63 ± 0.06 | | 5 |
| T128 | ScTSEN2 | | −1.62 ± 0.07 | | 9 |
| K328 | ScTSEN2 | | −1.44 ± 0.10 | | 9 |
| T123 | ScTSEN2 | | −1.29 ± 0.07 | | 6 |
| N7 | ScTSEN2 | | −1.21 ± 0.08 | | 7 |
| K368 | ScTSEN2 | | −1.20 ± 0.10 | | 7 |
| P294 | ScTSEN2 | | −1.11 ± 0.07 | | 9 |
| G325 | ScTSEN2 | | −1.08 ± 0.05 | | 7 |
| V127 | ScTSEN2 | | −1.02 ± 0.08 | | 6 |
| F138 | ScTSEN2 | | −1.00 ± 0.08 | | 8 |
| Q32 | ScTSEN34 | | −5.32 ± 0.09 | | 9 |
| K107 | ScTSEN34 | | −5.00 ± 0.10 | | 9 |
| K100 | ScTSEN34 | | −4.62 ± 0.09 | | * |
| V35 | ScTSEN34 | | −4.32 ± 0.08 | | 5 |
| R201 | ScTSEN34 | | −4.18 ± 0.11 | | * |
| F202 | ScTSEN34 | | −4.01 ± 0.09 | | 9 |
| T247 | ScTSEN34 | | −3.70 ± 0.07 | | 7 |
| K249 | ScTSEN34 | | −3.66 ± 0.10 | | * |
| R113 | ScTSEN34 | | −3.43 ± 0.11 | | 3 |
| L135 | ScTSEN34 | | −3.05 ± 0.08 | | 8 |
| R243 | ScTSEN34 | | −2.69 ± 0.11 | | 9 |
| I111 | ScTSEN34 | | −2.59 ± 0.09 | | 6 |
| V248 | ScTSEN34 | | −2.46 ± 0.08 | | 9 |
| H104 | ScTSEN34 | | −2.36 ± 0.08 | | 7 |
| Q33 | ScTSEN34 | | −2.34 ± 0.09 | | 9 |
| K110 | ScTSEN34 | | −1.90 ± 0.12 | | * |
| I136 | ScTSEN34 | | −1.87 ± 0.09 | | 6 |
| T29 | ScTSEN34 | | −1.84 ± 0.08 | | 6 |
| N132 | ScTSEN34 | | −1.72 ± 0.08 | | 5 |
| K250 | ScTSEN34 | | −1.67 ± 0.10 | | * |
| W271 | ScTSEN34 | | −1.45 ± 0.09 | | 9 |
| A30 | ScTSEN34 | | −1.35 ± 0.06 | | 9 |
| T246 | ScTSEN34 | | −1.35 ± 0.08 | | 9 |
| L214 | ScTSEN34 | | −1.15 ± 0.08 | | 9 |
| S139 | ScTSEN34 | | −1.10 ± 0.06 | | 8 |
| L108 | ScTSEN34 | | −1.05 ± 0.08 | | 6 |
| P213 | ScTSEN34 | | −1.04 ± 0.07 | | 9 |
| R50 | ScTSEN54 | | −8.60 ± 0.11 | | 9 |
| K458 | ScTSEN54 | | −7.89 ± 0.10 | | 1 |
| W464 | ScTSEN54 | | −6.08 ± 0.11 | | * |
| K108 | ScTSEN54 | | −5.34 ± 0.10 | | 8 |
| R307 | ScTSEN54 | | −5.29 ± 0.11 | | 9 |
| R405 | ScTSEN54 | | −5.16 ± 0.13 | | 2 |
| R416 | ScTSEN54 | | −4.96 ± 0.12 | | 5 |
| K49 | ScTSEN54 | | −4.86 ± 0.10 | | 8 |
| K459 | ScTSEN54 | | −4.60 ± 0.10 | | 5 |
| K305 | ScTSEN54 | | −4.53 ± 0.10 | | 9 |
| Q462 | ScTSEN54 | | −4.52 ± 0.09 | | 6 |
| K406 | ScTSEN54 | | −4.34 ± 0.11 | | 5 |
| K466 | ScTSEN54 | | −4.05 ± 0.11 | | * |
| K378 | ScTSEN54 | | −3.87 ± 0.13 | | 7 |
| K402 | ScTSEN54 | | −3.82 ± 0.10 | | 7 |
| S110 | ScTSEN54 | | −3.66 ± 0.07 | | 8 |
| N42 | ScTSEN54 | | −3.57 ± 0.08 | | 7 |
| Q113 | ScTSEN54 | | −3.47 ± 0.09 | | 8 |
| S120 | ScTSEN54 | | −3.45 ± 0.07 | | 5 |
| S302 | ScTSEN54 | | −3.45 ± 0.06 | | 5 |
| K394 | ScTSEN54 | | −3.29 ± 0.10 | | 3 |
| K306 | ScTSEN54 | | −3.26 ± 0.09 | | 9 |
| M112 | ScTSEN54 | | −3.22 ± 0.08 | | 7 |
| Y408 | ScTSEN54 | | −3.17 ± 0.09 | | 1 |
| A43 | ScTSEN54 | | −3.06 ± 0.06 | | 4 |
| K88 | ScTSEN54 | | −3.06 ± 0.10 | | 9 |
| N303 | ScTSEN54 | | −3.04 ± 0.08 | | 8 |
| R137 | ScTSEN54 | | −2.73 ± 0.11 | | 9 |
| K403 | ScTSEN54 | | −2.66 ± 0.11 | | 5 |
| A44 | ScTSEN54 | | −2.58 ± 0.06 | | 4 |
| I412 | ScTSEN54 | | −2.53 ± 0.10 | | 5 |
| Q457 | ScTSEN54 | | −2.34 ± 0.09 | | 3 |
| L47 | ScTSEN54 | | −2.26 ± 0.08 | | 7 |
| G109 | ScTSEN54 | | −2.23 ± 0.05 | | 9 |
| A118 | ScTSEN54 | | −2.23 ± 0.06 | | 6 |
| T401 | ScTSEN54 | | −2.10 ± 0.07 | | 4 |
| H301 | ScTSEN54 | | −2.10 ± 0.08 | | 7 |
| K465 | ScTSEN54 | | −2.03 ± 0.11 | | * |
| S46 | ScTSEN54 | | −1.96 ± 0.06 | | 5 |
| R375 | ScTSEN54 | | −1.89 ± 0.13 | | 1 |
| P300 | ScTSEN54 | | −1.81 ± 0.07 | | 9 |
| L45 | ScTSEN54 | | −1.81 ± 0.08 | | 5 |
| K53 | ScTSEN54 | | −1.70 ± 0.09 | | 9 |
| R463 | ScTSEN54 | | −1.68 ± 0.12 | | * |
| A377 | ScTSEN54 | | −1.54 ± 0.06 | | 5 |
| L189 | ScTSEN54 | | −1.53 ± 0.09 | | 9 |
| Q398 | ScTSEN54 | | −1.43 ± 0.09 | | 4 |
| K419 | ScTSEN54 | | −1.43 ± 0.11 | | 9 |
| P107 | ScTSEN54 | | −1.35 ± 0.07 | | 7 |
| F304 | ScTSEN54 | | −1.33 ± 0.08 | | 8 |
| N415 | ScTSEN54 | | −1.25 ± 0.08 | | 4 |
| K106 | ScTSEN54 | | −1.25 ± 0.11 | | 5 |
| D119 | ScTSEN54 | | −1.22 ± 0.08 | | 8 |
| G122 | ScTSEN54 | | −1.19 ± 0.05 | | 8 |
| K404 | ScTSEN54 | | −1.08 ± 0.11 | | 4 |
| R188 | ScTSEN54 | | −1.00 ± 0.12 | | 9 |

**Notes:** Protein-side residues with ΔG_res_ ≤ −1 kcal mol^−1^ are shown. Energy values are reported as mean ± SEM.

^†^ Residues are denoted by the single-letter amino acid code followed by the position number.

^‡^ Chain identifiers in parentheses apply to AtTSEN only because two identical copies of each subunit are present; chains A and C denote the contributing protomers.

^§^ ConSurf conservation grades range from 1 to 9, with higher scores indicating stronger evolutionary conservation; grades 1–3 indicate variable positions, 4–5 intermediate conservation, and 6–9 highly conserved positions; * indicates insufficient data.

**Abbreviations:** At, *Arabidopsis thaliana*; Hs, *Homo sapiens*; Sc, *Saccharomyces cerevisiae*; ΔG_res_, per-residue binding free energy; MMGBSA, molecular mechanics generalized Born surface area; pre-tRNA, precursor transfer RNA; SEM, standard error of the mean.
