# Supplementary material for: An (αβ)2 Architecture of the Arabidopsis thaliana tRNA Splicing Endonuclease Complex: Structural Modeling and Evolutionary Analyses
Source: Proteins. 2026 Jul 9;94(12):1913–26. doi: 10.1002/prot.70155 (PMC13728533; doi:10.1002/prot.70155)
Supplement: Supplementary file 3 [file PROT-94-1913-s003.docx]

**Table S5.** MMGBSA binding free-energy components for TSEN–pre-tRNA complexes.

| **Energy component** | **AtTSEN–pre-tRNA^Tyr^** | **HsTSEN–pre-tRNA^Arg^** | **ScTSEN–pre-tRNA^Phe^** |
| --- | --- | --- | --- |
| ΔE_vdW_ | −288.25 ± 0.26 | −536.35 ± 0.33 | −578.55 ± 0.29 |
| ΔE_EL_ | −566.11 ± 0.79 | −1269.83 ± 0.75 | −2742.82 ± 0.85 |
| ΔG_GB_ | +530.13 ± 0.74 | +1211.64 ± 0.68 | +2667.78 ± 0.81 |
| ΔE_SURF_ | −38.48 ± 0.03 | −70.15 ± 0.03 | −75.25 ± 0.02 |
| ΔG_GAS_ | −854.36 ± 0.92 | −1806.18 ± 0.85 | −3321.37 ± 0.89 |
| ΔG_SOLV_ | +491.65 ± 0.73 | +1141.49 ± 0.67 | +2592.54 ± 0.81 |
| **ΔG_bind_** | **−362.71 ± 0.30** | **−664.69 ± 0.36** | **−728.83 ± 0.28** |

**Notes:** All energy values are reported as mean ± SEM (kcal mol⁻¹). Abbreviations: MMGBSA, molecular mechanics generalized Born surface area; pre-tRNA, precursor transfer RNA; SEM, standard error of the mean; Δ_EEL_, electrostatic energy; Δ_ESURF_, nonpolar solvation energy; Δ_EvdW_, van der Waals energy; Δ_Gbind_, total binding free energy; Δ_GGB_, polar solvation energy; Δ_GGAS_, total gas-phase energy; Δ_GSOLV_, total solvation energy.
