# Supplementary material for: An (αβ)2 Architecture of the Arabidopsis thaliana tRNA Splicing Endonuclease Complex: Structural Modeling and Evolutionary Analyses
Source: Proteins. 2026 Jul 9;94(12):1913–26. doi: 10.1002/prot.70155 (PMC13728533; doi:10.1002/prot.70155)
Supplement: Supplementary file 4 [file PROT-94-1913-s004.docx]

**Table S1 Database identifiers of protein monomers and pre-tRNA sequences used in this study.**

| **Name** | **UniProt accession** | **GtRNAdb ID** |
| --- | --- | --- |
| AtTSEN1 | Q9M1E8 | — |
| AtTSEN2 | F4JYW0 | — |
| AtTSEN54 | Q6DBN3 | — |
| AtTSEN54 | Q9FWA2 | — |
| HsTSEN2 | Q8NCE0 | — |
| HsTSEN34 | Q9BSV6 | — |
| HsTSEN54 | Q7Z6J9 | — |
| HsTSEN15 | Q8WW01 | — |
| ScTSEN2 | P16658 | — |
| ScTSEN34 | P39707 | — |
| ScTSEN54 | Q02825 | — |
| ScTSEN15 | Q04675 | — |
| CeTSEN15 | A8HPJ9 | — |
| Pre-tRNA^Arg^ | — | chr17.trna3 |
| Pre-tRNA^Phe^ | — | chrVIII.trna7 |
| Pre-tRNA^Tyr^ | — | chr5.trna57 |

**Notes:** Organism prefixes: At, *Arabidopsis thaliana*; Ce, *Chlamydomonas eustigma*; Hs, *Homo sapiens*; Sc, *Saccharomyces cerevisiae.* Abbreviations: GtRNAdb, Genomic tRNA Database; pre-tRNA, precursor transfer RNA; UniProt, Universal Protein Resource.
