# Supplementary material for: An (αβ)2 Architecture of the Arabidopsis thaliana tRNA Splicing Endonuclease Complex: Structural Modeling and Evolutionary Analyses
Source: Proteins. 2026 Jul 9;94(12):1913–26. doi: 10.1002/prot.70155 (PMC13728533; doi:10.1002/prot.70155)
Supplement: Supplementary file 5 [file PROT-94-1913-s005.docx]

**Table S3.** Plasmids used for bimolecular fluorescence complementation analysis (BiFC).

| **Plasmids** | **References** |
| --- | --- |
| pB4cVGW | [34] |
| pB4GWcV | [34] |
| pB4GWnV3 | [34] |
| pB4nVGW3 | [34] |
| pB4cVGW-AtTSEN1 | This study |
| pB4GWcV-AtTSEN1 | This study |
| pB4GWnV3-AtTSEN1 | This study |
| pB4nVGW3-AtTSEN1 | This study |
| pB4cVGW-AtTSEN2 | This study |
| pB4GWcV-AtTSEN2 | This study |
| pB4GWnV3-AtTSEN2 | This study |
| pB4nVGW3-AtTSEN2 | This study |
| pB4cVGW-AtTSEN54 | This study |
| pB4GWcV-AtTSEN54 | This study |
| pB4GWnV3-AtTSEN54 | This study |
| pB4nVGW3-AtTSEN54 | This study |
| pDONR221 | [36] |
| pGWB45 | [35] |
