# Supplementary material for: An (αβ)2 Architecture of the Arabidopsis thaliana tRNA Splicing Endonuclease Complex: Structural Modeling and Evolutionary Analyses
Source: Proteins. 2026 Jul 9;94(12):1913–26. doi: 10.1002/prot.70155 (PMC13728533; doi:10.1002/prot.70155)
Supplement: Supplementary file 6 [file PROT-94-1913-s006.docx]

**Table S2.**  Primers used for cloning in this study

| **Name** | **Sequence from 5′ to 3′** | **References** |
| --- | --- | --- |
| attB1_F | GGGGACAAGTTTGTACAAAAAAGCAGGCT | [36] |
| attB2_R | GGGGACCACTTTGTACAAGAAAGCTGGGT | [36] |
| AtTSEN1_GW_F | AAAAAGCAGGCTCCATGGCACCAAGGTGGA | This study |
| AtTSEN1_GW_R | AGAAAGCTGGGTATGTTCTGGTTTCATCTT | This study |
| AtTSEN2_GW_F | AAAAAGCAGGCTCCATGGCGCCTAGATGGA | This study |
| AtTSEN2_GW_R | AGAAAGCTGGGTAAAGAGTGTTCAAATTAC | This study |
| AtTSEN54_GW_F | AAAAAGCAGGCTTTATGGAGGAAAAAGACT | This study |
| AtTSEN54_GW_R | AGAAAGCTGGGTATGATAAGACAGGGAGGT | This study |
