# Supplementary material for: An (αβ)2 Architecture of the Arabidopsis thaliana tRNA Splicing Endonuclease Complex: Structural Modeling and Evolutionary Analyses
Source: Proteins. 2026 Jul 9;94(12):1913–26. doi: 10.1002/prot.70155 (PMC13728533; doi:10.1002/prot.70155)
Supplement: Supplementary file 7 [file PROT-94-1913-s007.docx]

**Supporting Information**

**An (αβ)₂ Architecture of the Arabidopsis thaliana tRNA Splicing Endonuclease Complex: Structural Modeling and Evolutionary Analyses**

**Authors:** Mohammad Moniruzzaman^1^, Markus Englert^3^, Kazuhito Akama^1,2^*

**Affiliations:**

^1^ Graduate School of Natural Science & Technology, Shimane University, 1060 Nishikawatsu, Matsue, Shimane 690-8504, Japan.

^2^ Department of Life Sciences, Shimane University, 1060 Nishikawatsu, Matsue, Shimane 690-8504, Japan.

^3^ Independent researcher; current affiliation: Constructive.Bio, Whittlesford, UK.

***Corresponding author:** Prof. Kazuhito Akama

**Email:** [akama@life.shimane-u.ac.jp](mailto:akama@life.shimane-u.ac.jp)

**Figure S1.** Multiple sequence alignment of putative AtTSEN54 homologs from *Arabidopsis thaliana*. Sequences were aligned using Clustal Omega version 1.2.4. Columns are colored with the ClustalX scheme. Conservation symbols: “*” fully conserved; “:” strongly similar substitution; “.” weakly similar substitution; “-” gap. Organism prefix: At, *Arabidopsis thaliana*.

**Figure S2.** Multiple sequence alignment of TSEN54 homologs from *Arabidopsis thaliana*, *Homo sapiens*, and *Saccharomyces cerevisiae*. Sequences were aligned using Clustal Omega version 1.2.4. Columns are colored according to the ClustalX scheme. Organism prefixes: At, *Arabidopsis thaliana*; Hs, *Homo sapiens*; Sc, *Saccharomyces cerevisiae*.

**Figure S3.** Bimolecular fluorescence complementation (BiFC) analysis of AtTSEN1–AtTSEN2 interactions. (A) Representative confocal micrographs of onion epidermal cells. For each panel, columns show the internal ECFP control channel (magenta), the BiFC Venus channel (green), and the merged overlay. Scale bars, 50 µm. A non-interacting vector pair (pB4cVGW/pB4nVGW3) served as the negative control. (B) Quantification of BiFC signal intensity, expressed as the Venus/ECFP fluorescence ratio. Bars indicate mean ± SEM (*n* = 4 cells per condition). No statistically significant difference versus the negative control was detected by one-way ANOVA followed by Dunnett’s post hoc test (*p* = 0.578; **p* ≤ 0.05; ***p* ≤ 0.01; ****p* ≤ 0.001). In panel labels, vector names omit the pB4 prefix; protein names are abbreviated as S1 (AtTSEN1), S2 (AtTSEN2), and S54 (AtTSEN54). Abbreviations: ANOVA, analysis of variance; ECFP, enhanced cyan fluorescent protein; SEM, standard error of the mean; µm, micrometer.

**Figure S4.** Confidence assessment of AlphaFold3 (AF3) TSEN complex models generated in this study. Each panel shows the top-ranked (rank-0) AF3 model colored by per-residue pLDDT (left) and the corresponding PAE heatmap (right): (A) AtTSEN complex; (B) AtTSEN–pre-tRNA^Tyr^; (C) HsTSEN–pre-tRNA^Arg^; and (D) ScTSEN–pre-tRNA^Phe^. pLDDT color scale: blue, very high confidence (pLDDT > 90); cyan, confident (70–90); yellow, low confidence (50–70); red, very low confidence (<50). PAE heatmaps range from 0 to 30 Å; darker colors indicate lower expected positional error and higher inter-residue confidence, plotted as scored-residue index (x-axis) versus aligned-residue index (y-axis). pTM and ipTM scores are indicated within each panel; pTM > 0.5 indicates a reliable global fold, ipTM > 0.8 indicates a confident predicted interface, ipTM values of 0.6–0.8 indicate intermediate confidence, and ipTM < 0.6 indicates low confidence. Abbreviations: AF3, AlphaFold3; Å, ångström; ipTM, interface predicted template modeling score; PAE, predicted aligned error; pLDDT, predicted local distance difference test; pre-tRNA, precursor transfer RNA; pTM, predicted template modeling score.

**Figure S4 (continued)**
